# Ensemble frequency ratio-maximum entropy landslide susceptibility modelling for climate‑resilient road corridors in Bocas del Toro and Chiriquí, Panama

**DOI:** 10.1371/journal.pone.0362084

**Published:** 2026-11-05

**Authors:** Dereck Mbeh Petiangma, Gerald G. Singh, Enrique Williams, Jeffrey Gerardo Ramos Perez, Rachel Collin

**Affiliations:** 1 Smithsonian Tropical Research Institute, Bocas Research Station, Balboa Ancon, Panama; 2 Disaster Management Training and Education Center for Africa (DiMTEC), University of the Free State, Bloemfontein, South Africa; 3 School of Environmental Studies, University of British Columbia, Vancouver, British Columbia, Canada; 4 Centro Regional de Bocas del Toro, Universidad de Panama, Panamá; 5 Centro Regional de Bocas del Toro, Universidad Tecnologica de Panama, Panamá; Hanoi University of Mining and Geology, VIET NAM

## Abstract

Landslides are a major rainfall-triggered hazard affecting road infrastructure across the mountainous terrain of western Panama. This study develops an ensemble Frequency Ratio (FR)-Maximum Entropy (MaxEnt) modelling framework to assess landslide susceptibility, identify critical management zones, and determine key drivers of slope failure along road corridors in the mainland districts of Bocas del Toro and along the Rambala-Hornito-David route. Within a 1,000 m corridor buffer, 87 mapped landslides (63 for training and 24 for validation) and 15 environmental conditioning factors were used to develop and validate the FR and MaxEnt models. An AUC-weighted linear combination of the independently calibrated FR and MaxEnt models was used to generate the ensemble susceptibility map. The ensemble delineated 9.67% and 3.99% of the corridor as high and very high susceptibility, respectively, achieving the highest predictive performance (AUC = 0.945) and outperforming the FR (0.873) and MaxEnt (0.943) models. Sensitivity analyses based on alternative training-validation partitions (57:30 and 70:17) produced consistently good performance (prediction-rate AUC > 0.88), indicating that the principal findings were not strongly influenced by the inventory allocation. Hotspot analysis identified three major clusters of elevated susceptibility and confirmed statistically significant spatial clustering at the 95% and 99% confidence levels. More than 71 km of primary roads intersect high-susceptibility zones, with Hornito, Bajo Cedro, and Valle de Agua Arriba exhibiting the greatest exposure. Slope gradient and NDVI were identified as the most influential predictors. These findings provide a practical basis for prioritising monitoring, slope stabilisation, drainage improvement, and ecosystem-based interventions to strengthen the resilience of road infrastructure in tropical mountain environments. Future work should incorporate larger landslide inventories, event-based rainfall information, and climate-change scenarios to improve the representation of landslide dynamics and support risk-informed infrastructure planning.

## 1 Introduction

Landslides are widespread and destructive hazards that threaten settlements, transportation corridors, and ecosystems worldwide [1]. Global disaster trends reported under the Sendai Framework for Disaster Risk Reduction and the 2030 Agenda for Sustainable Development highlight the need to strengthen infrastructure resilience, with reported disasters increasing from an annual average of 371 - between 2004–2023 to 393 in 2024 [2]. Landslides and related geohazards cause thousands of deaths each year and contribute substantially to global economic losses, estimated at more than 2.3 trillion U.S. dollars [3,4].

Climate change is expected to intensify landslide risk by altering precipitation regimes and increasing the frequency of extreme rainfall events, especially in mountainous tropical regions [1,5]. Globally, more than 27,000 km of roads and 6,000 km of railways lie within high landslide-hazard zones, making transport networks among the infrastructure systems most exposed to slope instability [6]. As climate-resilient development gains policy attention, predictive models that incorporate present and future climate conditions are increasingly important for risk-sensitive infrastructure planning and mitigation [7–9].

In Central America, rugged topography, intense rainfall, and active tectonics make landslides a persistent regional hazard [10,11]. The most severe events are often associated with tropical cyclones and prolonged rainfall [12]. For example, Hurricanes Eta and Iota triggered widespread slope failures in Guatemala and Honduras in 2020, causing hundreds of deaths and extensive infrastructure damage [13–15]. Between 2004 and 2013, landslides associated with earthquakes and sustained rainfall caused 11,631 fatalities across Latin America and the Caribbean [16]. Regional climate analyses indicate that increases in extreme rainfall are likely to intensify landslide occurrence [17,18], while impacts on transport networks and other lifelines have been documented in Costa Rica [19]. Despite these risks, landslide research remains uneven across Latin America and the Caribbean [16]. This study responds to that gap by linking landslide susceptibility to road-infrastructure exposure to support risk-sensitive planning.

Panama lies at the climatic and geological crossroads of Central America [20] and faces increasing risk from rainfall-induced landslides, the country’s most common flood-related secondary hazard [21]. Between 1933 and 2019, 625 landslide events were recorded nationally [22], and official reports indicate continued activity from 2019 to 2024, including 74 events in 2021 alone [23]. Landslides are especially prevalent in the western provinces of Bocas del Toro and Chiriquí and in the Comarca Ngäbe-Buglé, where recurrent slope failures have been reported [21,24,25]. In 2020, Hurricanes Eta and Iota triggered severe flooding and landslides across these provinces, damaging roads, bridges, and homes [26]. Because Panama has one of the lowest road densities in Central America [27], landslide-related road disruptions can strongly affect trade, mobility, and access to essential services. These vulnerabilities underscore the need for advanced, climate-informed susceptibility modelling to guide infrastructure planning and resilience-building [5,8].

Landslide susceptibility mapping has evolved from heuristic assessments to statistically grounded and machine-learning approaches [28,29]. Logistic regression remains widely used because it is transparent and interpretable [30,31], although its performance can be constrained by multicollinearity and by categorical predictors such as lithology [28]. The Frequency Ratio (FR) method, while limited in its ability to capture nonlinear interactions, remains useful in data-scarce mountainous regions because it is simple and interpretable [32–35]. Maximum Entropy (MaxEnt), originally developed for ecological niche modelling, is increasingly used in landslide studies because it performs well with presence-only datasets, a common limitation of landslide inventories [36–39]. Recent studies also show growing use of ensemble and hybrid frameworks to improve predictive robustness [40–43]. However, weighted FR–MaxEnt ensembles remain uncommon in the accessible literature. This study applies an integrated FR–MaxEnt ensemble to map landslide susceptibility along climate-critical road corridors in western Panama, identify vulnerable road segments, and determine the environmental controls on susceptibility. By doing so, it extends ensemble geospatial modelling to a data-limited tropical infrastructure context where corridor-scale susceptibility evidence remains scarce.

## 2 Geographical setting and method

### 2.1 Geographical setting

This study evaluates landslide susceptibility along major road corridors in mainland Bocas del Toro Province and the Rambala–Hornito–David route, the region’s main terrestrial link to the rest of Panama. These corridors are repeatedly affected by rainfall-triggered slope failures. In 2008, severe floods and landslides in the area destroyed highways and bridges, isolating several communities [44]. In 2021, intense rainfall in the area triggered landslides that blocked access to Charco La Pava, Valle de Rey, Bajo Esperanza, and the Chiriquí Grande–Hornito–David corridor [25,45]. More recently, mud and debris flows obstructed the Chiriquí Grande–Gualaca highway on 3 August 2025, again disrupting mobility and underscoring the road network’s persistent vulnerability [46]. These disruptions threaten regional economic resilience, particularly tourism, which depends on reliable transport infrastructure [47]. Although national assessments acknowledge these risks, corridor-scale susceptibility analyses remain limited. Existing studies in Bocas del Toro and Chiriquí have focused mainly on broader regional or local mapping [24,48,49].

The analysis was conducted within a 1,000 m buffer on either side of the road corridors in the study area (Fig 1). This buffer width was selected to capture the terrain most likely to influence or be affected by road‑related slope instability. Although a formal sensitivity analysis of alternative buffer distances was not undertaken, the 1,000 m extent aligns with recent landslide susceptibility studies that applied comparable spatial delimitations for road‑adjacent terrain [50–52]. These studies typically employed buffer widths between 500 m and 1,000 m to encompass both immediate and proximal slope‑failure zones.

**Fig 1:**
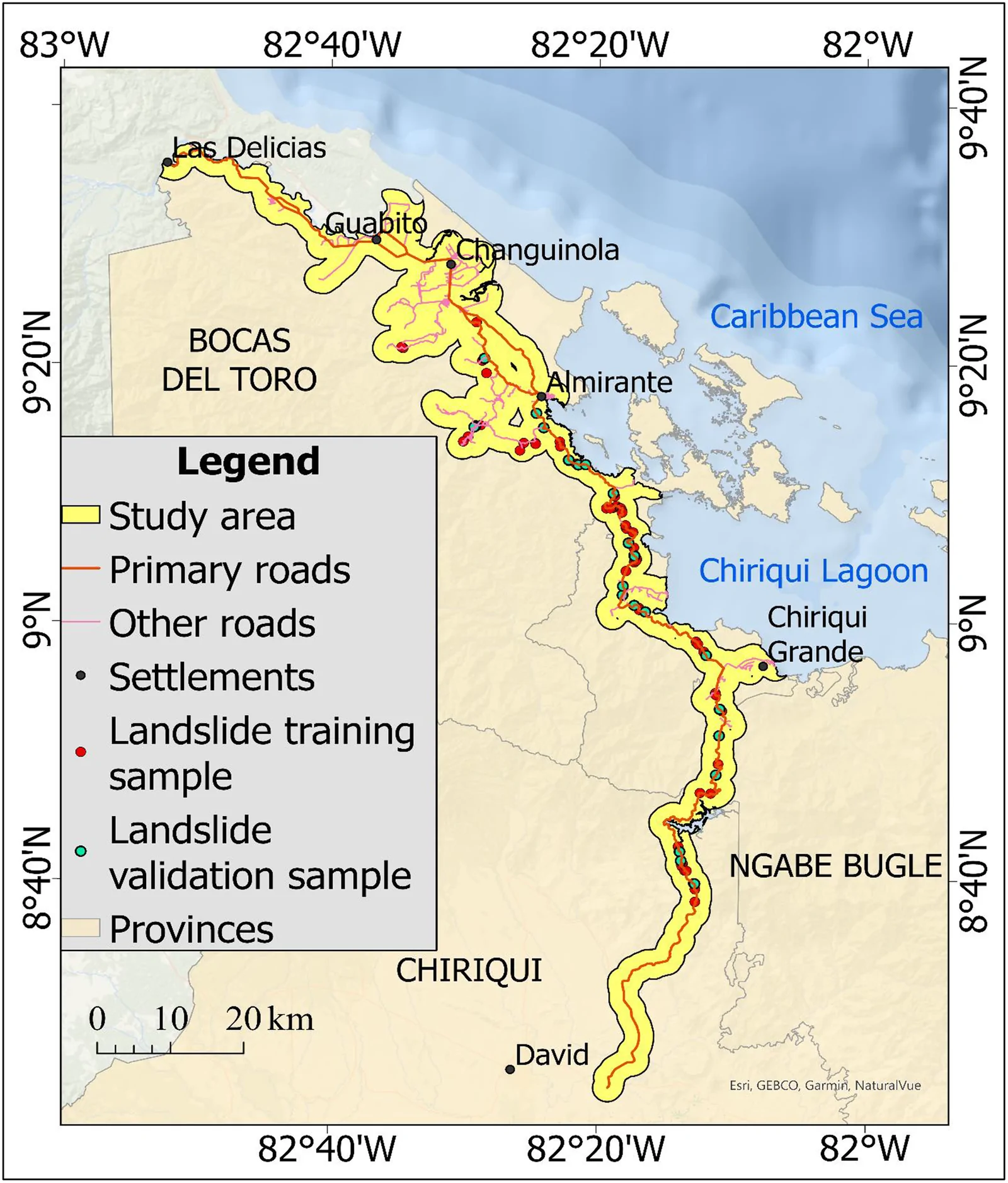
Location of the study area in Western Panama.

The island district of Bocas del Toro was excluded from the analysis because it lacks continuous terrestrial transport infrastructure. The buffered road network used in the study was derived from Panama’s 2018 national road vector dataset, processed from OpenStreetMap and hosted in the Smithsonian GIS repository [53]. To improve positional accuracy, the 2024 OpenStreetMap dataset was consulted to correct misaligned segments and digitise missing primary and secondary roads.

The 1,231.93 km^2^ study area spans elevations from 5 m in the coastal lowlands to 1,997 m in the highlands of Hornito, with steep slopes (> 19.8°) dominating the upland terrain (Fig 4a–4b). The coastal lowlands are underlain by sedimentary units—including alluvium, mangrove deposits, corals, and coal shale—whereas the highlands are characterised by volcanic rocks such as andesite, basalt, and ash (Fig 6c). The region lies within the Tropical Rainforest (Af) climatic zone, marked by a bimodal rainfall regime with a brief mid‑summer dry period and a secondary precipitation maximum from November to December [54–56]. Monthly temperatures range from 25.3 to 27.3 °C, while rainfall varies from 172 mm in October to 462 mm in July [57].

The study was conducted under the research authorisation issued by the Panama Ministry of Culture (Ministerio de Cultura) for the broader project hosted at the Smithsonian Tropical Research Institute (STRI). The analysis relied exclusively on publicly accessible road corridors and non-intrusive geospatial datasets; therefore, no additional site-specific permits were required. Drone-based imagery which show sample landslide scars were obtained through collaborators at the branch of the University of Panama in Changuinola, under their institutional flight permission.

### 2.2 Methods

An AUC-weighted FR–MaxEnt ensemble model was employed to assess landslide susceptibility. The primary workflow (Fig 2) integrated data preparation, multicollinearity diagnostics, model calibration using 72.4:27.6% training–validation partition, ensemble generation, hotspot identification, road-exposure assessment, and variable-importance analysis. A separate sensitivity analysis detailed in section 2.2.6 evaluated whether model performance was robust to alternative training–validation partitions.

**Fig 2:**
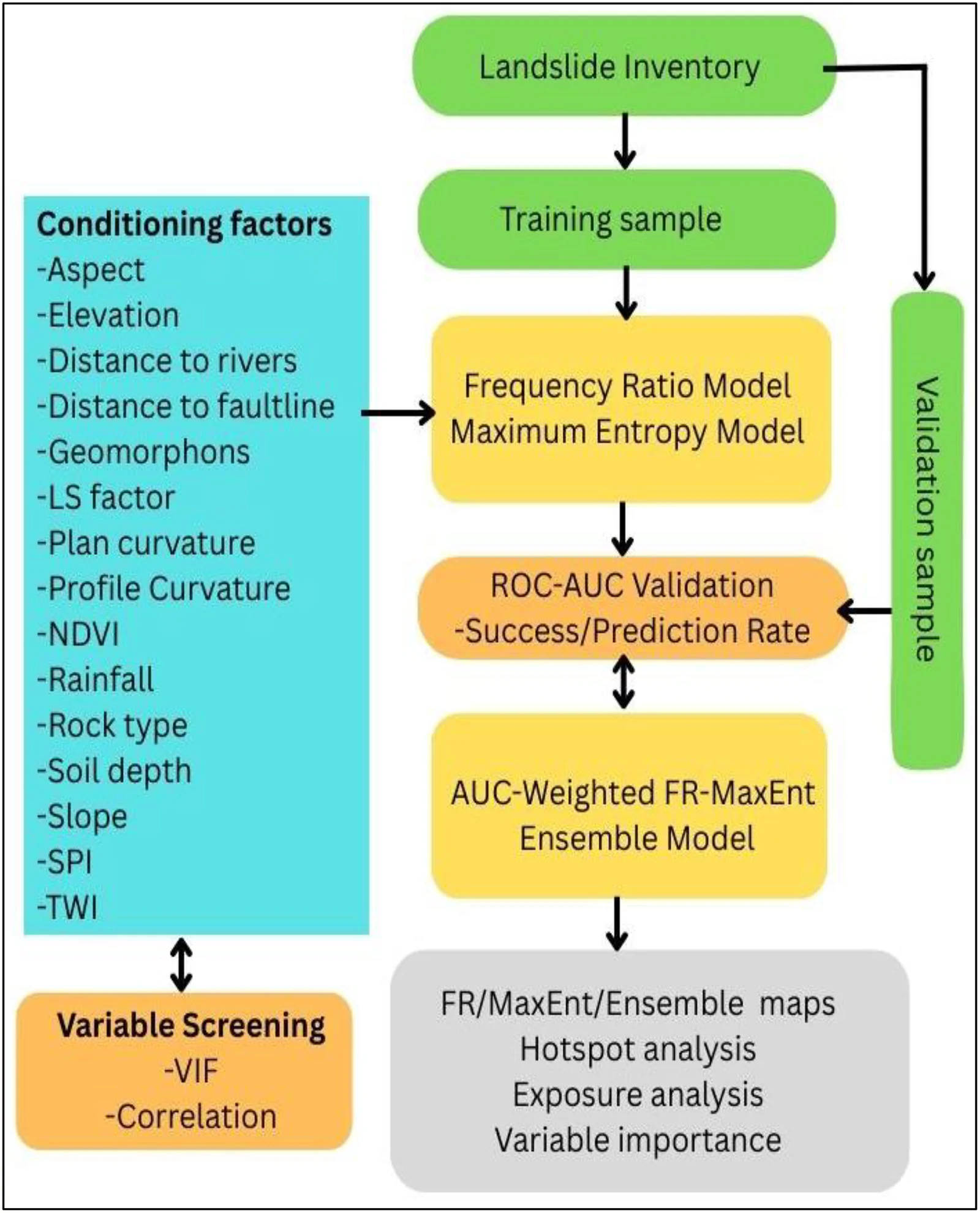
Flow chart of the research methodology. NB. LS-factor (slope length and steepness factor), SPI (stream power index), TWI (topographic wetness index), NDVI (normalize different vegetation index).

#### 2.2.1 Landslide inventory.

A total of 87 landslide scars were mapped along the entire road corridor through direct field observation using a Garmin GPS unit (S1 Table), as no formal landslide inventory exists for the region. This sample size is consistent with numerous corridor‑based susceptibility studies that report fewer than 90 mapped failures due to the narrow spatial extent and limited accessibility of linear infrastructure [58,59]. Field mapping enabled the identification of both large and small, including partially vegetated, failures that are often undetectable in satellite imagery, providing a complete and representative set of observable landslides for the study. For the Frequency Ratio (FR) model, 63 scars (72.4%) were used for calibration and 24 scars (27.6%) for validation (Fig 3), consistent with widely applied 70–75% and 25–30% splits [60,61]. In contrast, the MaxEnt model followed presence‑only practice, using the same training sample together with randomly sampled environmental background points (Section 2.2.4) drawn from the same accessible terrain to reduce spatial sampling bias and ensure comparable sampling effort. Unmapped areas were therefore treated as environmental background rather than true absences [38,39]. Although no random seed was specified, the primary FR, MaxEnt, and ensemble models were all developed using the same 63:24 training-validation partition to ensure consistent model comparison. The alternative partitions described in section 2.2.6 were used solely for sensitivity analysis.

**Fig 3:**
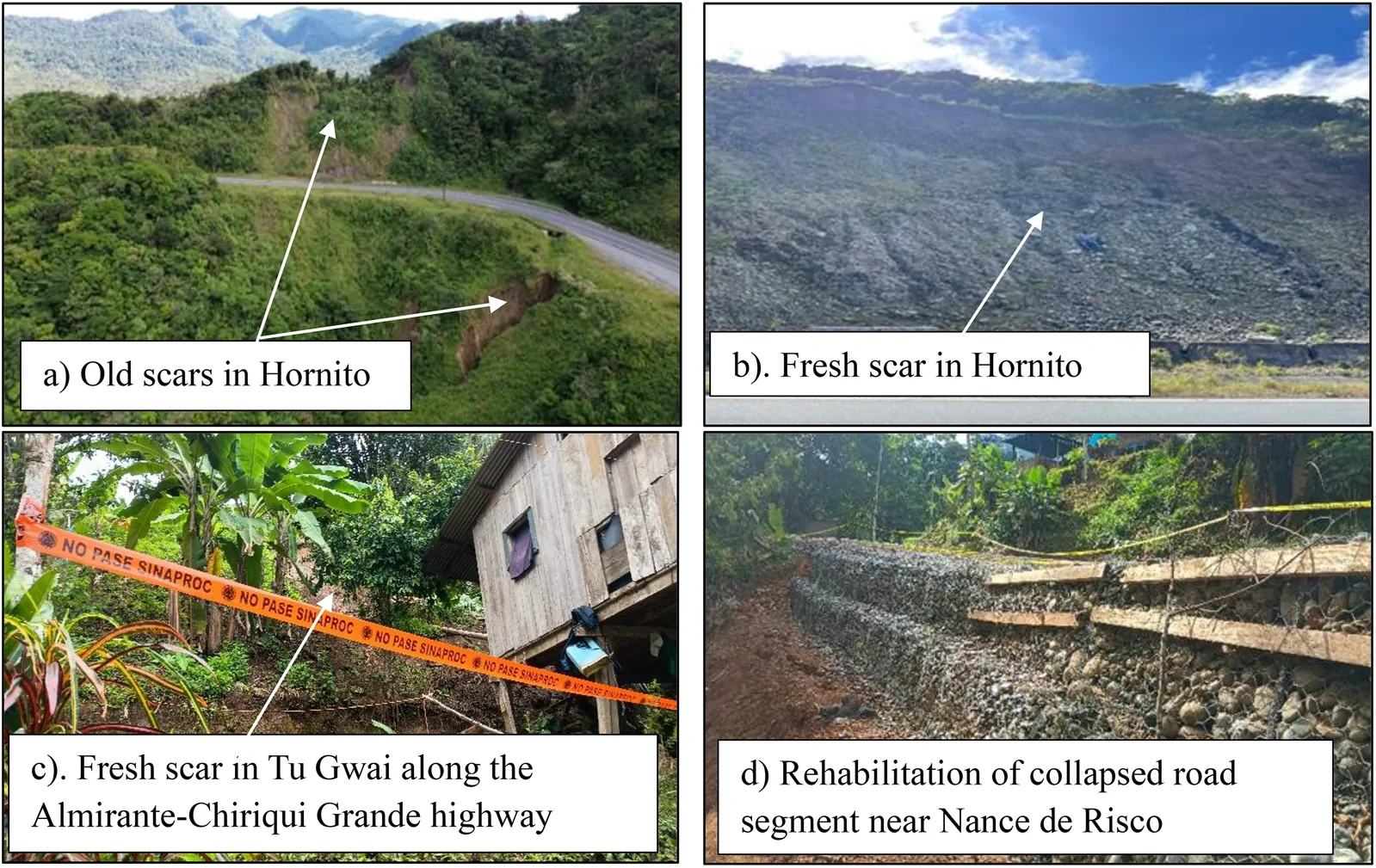
Landslide scars along road corridors in Hornito, Tu Gwai, and Nance de Risco.

#### 2.2.2 Conditioning factors.

Fifteen conditioning factors were selected based on established landslide research in tropical and subtropical environments [60,62–65] and the physical geography of the study area. These variables were grouped into four categories: terrain, hydrological, environmental, and geological. Table 1 presents the data sources and geospatial datasets from which the fifteen conditioning variables were derived.

**Table 1:** Sources and attributes of geospatial data from which conditioning factors were derived.

Data description	Resolution	Source
ALOS PALSAR DEM	12.5 m	https://doi.org/10.5067/Z97HFCNKR6VA
Interpreted Faultline of Panama	1: 250,000	[66] https://stridata-si.opendata.arcgis.com/datasets/SI::interpreted-fault-lines-of-panama/about
Geology of the Republic of Panama	1: 250,000	[67] https://stridata-si.opendata.arcgis.com/datasets/SI::geology-of-the-republic-of-panama-1250000-stri-digitized/about
Soil depth (SoilGrids250m)	250 m	[68] https://files.isric.org/soilgrids/former/2017-03-10/data/
Sentinel-2 quarter 1 mosaic of 2025	10 m	https://browser.dataspace.copernicus.eu/
90^th^ percentile historic rainfall for Central America	1 km	[69]

All datasets were harmonised by resampling to a common spatial resolution of 12.5 m, matching the ALOS PALSAR DEM from which terrain and hydrological variables were derived, and subsequently projected to UTM Zone 17N (WGS 1984). The DEM was hydrologically corrected prior to deriving terrain and hydrological variables. Continuous variables were reclassified for the FR model but retained in their original form for MaxEnt. GIS processing and raster harmonisation were performed in ArcGIS Pro 3.6. FR and MaxEnt modelling within ArcGIS Pro were implemented using the Spatial Analyst tools and the embedded MaxEnt module. Because the ArcGIS‑embedded MaxEnt does not support jackknife variable‑importance evaluation, the analysis was conducted separately using the standalone MaxEnt software (version 3.4.4).

**Terrain variables**

Terrain variables—including slope, aspect, geomorphons, profile curvature, plan curvature, and elevation—were derived from the 12.5 m ALOS PALSAR DEM (Fig 4), using the relevant tools in the ArcGIS Pro Spatial Analyst toolbox.

**Fig 4:**
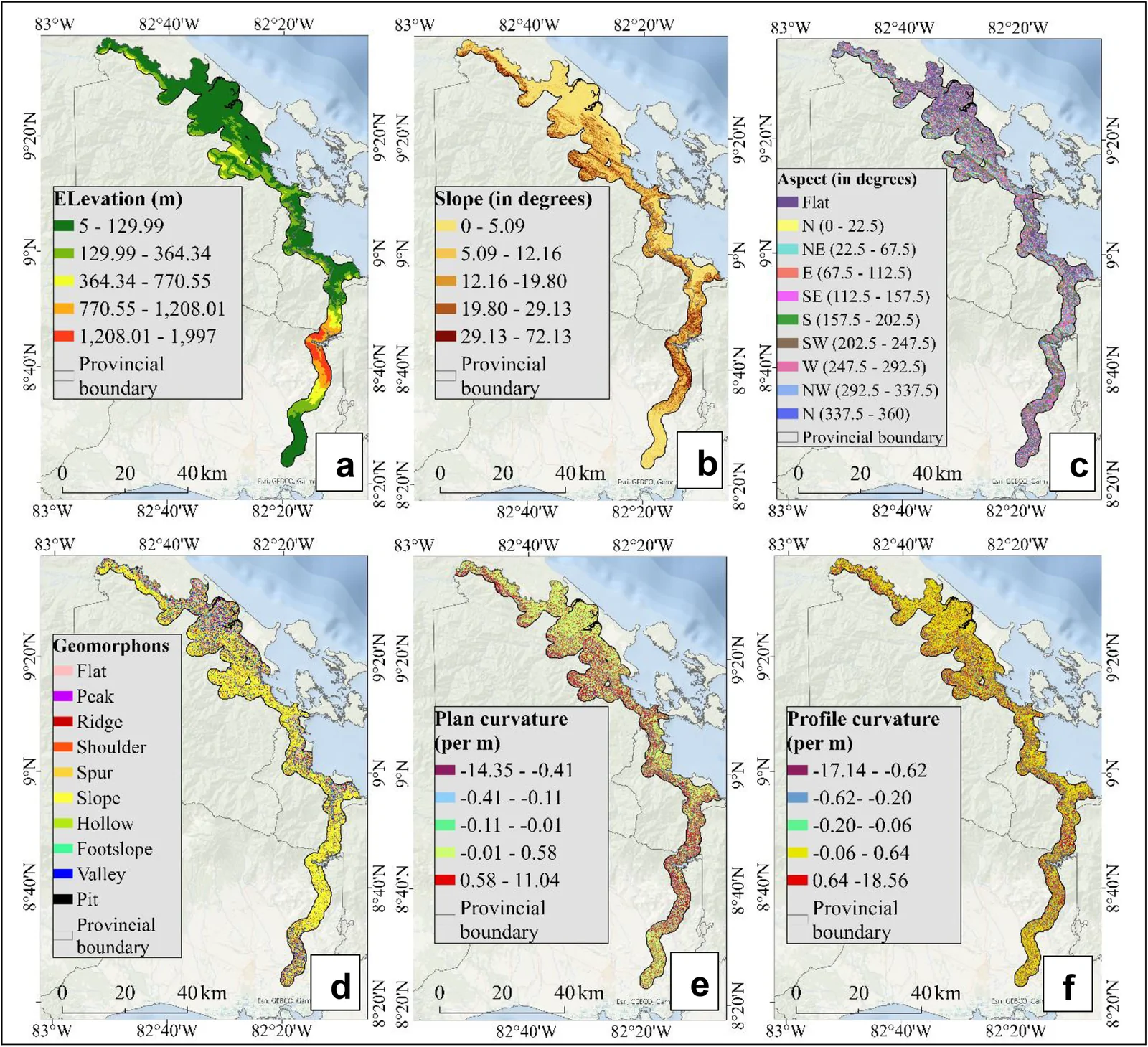
Terrain derived morphometric variables.

Steep slopes and high elevations are associated with increased susceptibility due to gravitational forces and orographic rainfall enhancement [30,70,71]. Elevation in the area ranges from 5 m to 1,997 m above sea level (Fig 4a), with steeper gradients concentrated at higher altitudes (Fig 4b). Slope and spur landforms dominate the area, accounting for 37.18% and 15.14% of the landscape, respectively (Fig 4d; Table 3). Curvature metrics (Figs 4e–4f) are predominantly positive, indicating divergent terrain, with convex, concave, and foot‑slope positions commonly associated with landslide initiation, rotational failures, and material accumulation, respectively [72,73].

**Hydrological variables**

Hydrological variables exert a fundamental influence on landslide initiation [74] and strongly shape the acceleration and failure mechanisms of rainfall‑triggered landslides [75]. Four hydrological predictors—Topographic Wetness Index (TWI), LS‑factor, Stream Power Index (SPI), and distance to rivers—were derived from the DEM (Fig 5). LS‑factor (Fig 5a) represents sediment‑transport potential [76], while SPI (Fig 5b) reflects the erosive power of concentrated flow [77]. TWI (Fig 5c) identifies zones of soil‑moisture convergence [78]. Distance to rivers (Fig 5d) was computed using Euclidean distance from flow‑accumulation‑derived channels, delineated using a threshold of 3,000 contributing cells.

**Fig 5:**
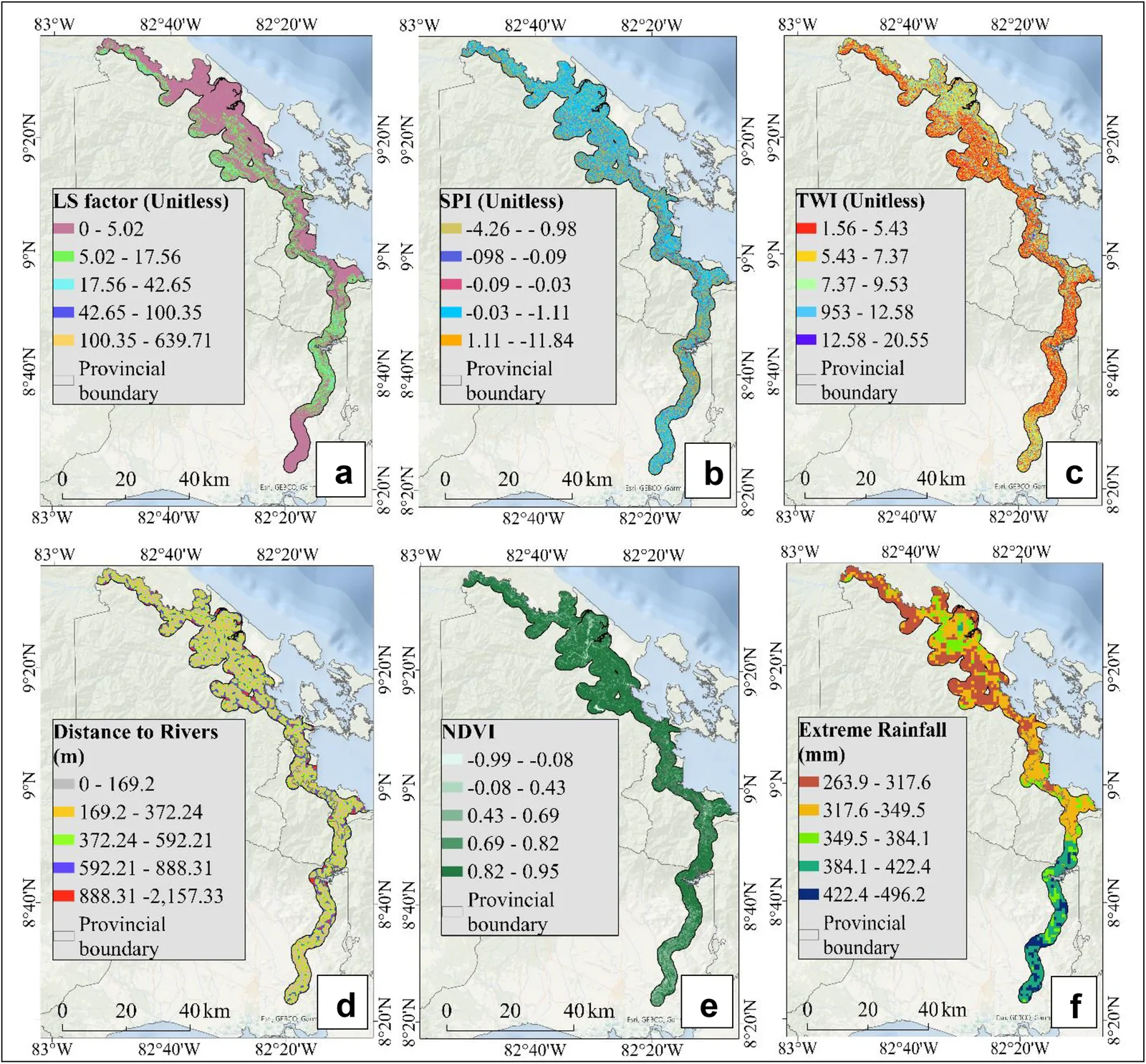
Hydrological and environmental variables.

Equations 1–3 were applied to compute TWI, LS‑factor, and SPI. The study area is characterised by low LS‑factor values, high SPI values, and low TWI values in upland zones.


$$\begin{array}{c} TWI=ln\left(\frac{{FA}_{scaled}}{tan\left(slope\right)}\right) \end{array}$$ (1)

Equation 1 follows the standard TWI formulation of Beven and Kirkby (78), where *ln* denotes the natural logarithm while *FA* represents flow accumulation. Scaled flow accumulation was computed as *(FA + 1) x 12.5*. Prior to that, slope in degrees was converted to radians (*slope in degrees x 1.570796)/ 90*, and a minimum tangent value of 0.001 was applied to avoid undefined values.


$$\begin{array}{c} {LS}_{factor}=\left(\frac{FA\times \text{1}\text{2}.\text{5}}{{\text{2}\text{2}.\text{1}\text{3}}^{m}}\right)\times \left(\frac{sin\left(slope\times \text{0}.\text{0}\text{1}\text{7}\text{4}\text{5}\right)}{{\text{0}.\text{0}\text{8}\text{9}\text{6}}^{n}}\right) \end{array}$$ (2)

Equation 2 represents a modified LS‑factor formulation derived from the Revised Universal Soil Loss Equation (RUSLE). Here, *FA* represents flow accumulation, 12.5 is the DEM resolution (m), 22.13 m is the standard RUSLE slope‑length unit, and 0.01745 converts slope from degrees to radians. The reference steepness value 0.0896 and empirical exponents *m* and *n* follow Moore and Burch [76] which typically range from between 0.4–0.5 and 1.2–1.3, respectively. Accordingly, this study adopted *m* = 0.4 and *n* = 1.3.


$$\begin{array}{c} SPI=ln[FA\text{\textasciitilde{}}x\text{\textasciitilde{}}Cell\text{\textasciitilde{}}Size)x\text{\textasciitilde{}}tan(Slope\text{\textasciitilde{}}in\text{\textasciitilde{}}degrees\text{\textasciitilde{}}x\text{\textasciitilde{}}0.01745)] \end{array}$$ (3)

Equation 3 follows the standard SPI formulation of Moore and Burch [76], where *ln* denotes the natural logarithm, *FA* represents flow accumulation, *tan* expresses slope steepness, and 0.01745 converts slope from degrees to radians.

Recent empirical studies indicate that the LS‑factor and SPI are positively correlated with landslide occurrence, whereas TWI frequently exhibits a negative association with shallow failures [79]. The study area is dominated by low LS‑factor values (< 17.56), high SPI values (> –0.03), and low TWI values in the highland zones. Also, over 58% of the area is at proximity (< 375 m) to rivers (Figs 5a-5d; Table 3).

**Environmental variables**

Environmental predictors were NDVI and extreme rainfall. NDVI was computed from the 2025 Sentinel‑2 Quarter 1 mosaic (Equation 4). More than 90% of the landscape exhibited NDVI values above 0.69, with lower values concentrated in settlements and cultivated areas (Fig 5e; Table 3).


$$\begin{array}{c} NDVI=\frac{Band\;\text{8}-Band\;\text{4}}{Band\;\text{8}+Band\;\text{4}} \end{array}$$ (4)

Extreme rainfall was derived from the 90th‑percentile historical rainfall dataset for Central America [69]. Values in the study area ranged from 264 mm in the coastal districts to more than 490 mm in the Hornito highlands (Fig 5f). Although dense forest vegetation contributes to slope stability, the frequency of shallow landslides increases with rainfall intensity and duration [80].

**Geological variables**

Geological predictors included rock type, soil depth, and distance to faults (Fig 6). Rock type and fault lines were extracted from the 1:250,000 geological map and interpreted fault‑line vector layers of Panama, while soil depth was obtained from the SoilGrids 250 m product [68]. Distance to faults was generated using the Euclidean distance tool applied to the interpreted fault‑line dataset. Landscapes characterised by active faulting, thin soils, and weak lithologies are generally more prone to slope failures [79,81,82].

**Fig 6:**
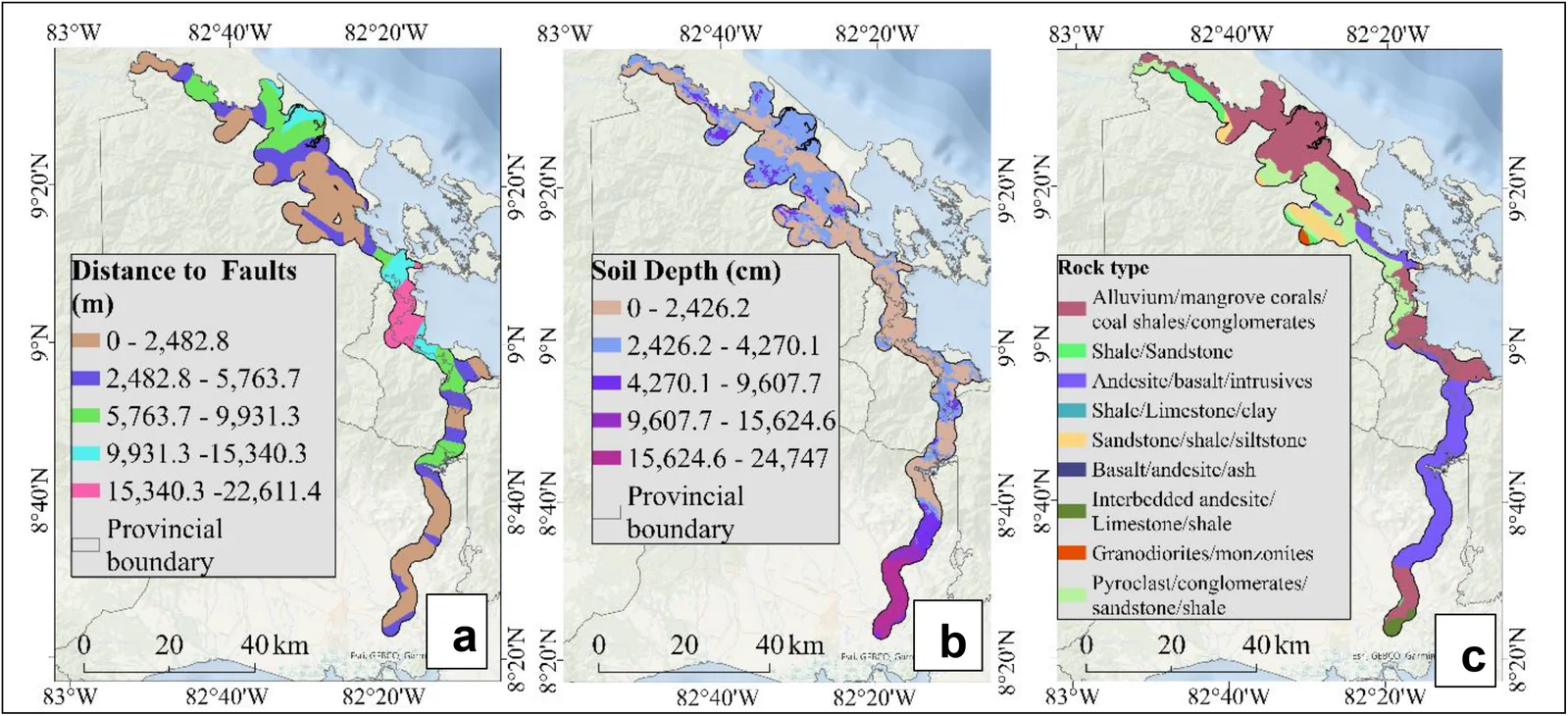
Geological and structural variables.

Proximity to major fault lines is lowest in the highlands around Hornito, Nance de Risco, and parts of Almirante Bay, but highest in the subdistrict of Punta Robalo in Chiriquí Grande (Fig 6a). Soil depth varies markedly across the region, ranging from 0 m in the highlands to more than 247 m in the lowlands of Chiriquí Province (Fig 6b). The geological framework comprises metamorphic, sedimentary, and volcanic units (Fig 6c).

#### 2.2.3 Screening of conditioning variables.

Multicollinearity among the conditioning factors was assessed using tolerance (Tol), Variance Inflation Factor (VIF), and pairwise Pearson correlation coefficients (r) (Table 2).

**Table 2:** Multicollinearity and correlation matrix for condition factors.

	Tol	VIF	D. Fl	Rt	SoD	Rain	NDVI	PrC	PlC	TWI	TRI	SPI	Sl	LSF	Asp	DR	Ele	Geo
D. Fl	.4	2.5	1															
Rt	.7	1.5	−.2	1														
SoD	.7	1.5	−.4	.1	1													
Rain	.3	3.5	−.2	−.4	.2	1												
NDVI	.8	1.3	.1	.0	.0	−.1	1											
PrC	.8	1.3	.1	−.2	−.2	.1	−.1	1										
PlC	.5	1.9	−.2	.2	.1	−.1	−.1	−.3	1									
TWI	.4	2.7	.3	−.0	−.0	.0	.2	.1	−.3	1								
TRI	.1	9.5	−.1	−.0	−.0	−.0	−.0	.2	−.1	−.5	1							
SPI	.7	1.5	.2	−.1	.0	.1	−.1	.2	−.4	.3	.0	1						
Sl	.1	9.1	−.1	−.0	.1	.1	−.1	.1	−.1	−.5	.9	.0	1					
LSF	.3	3.0	.1	.0	−.0	.1	.1	.3	−.5	.1	.6	.4	.6	1				
Asp	.7	1.4	−.4	.2	.2	−.0	−.0	−.1	.2	−.3	.2	−.0	.1	−.1	1			
DR	.7	1.4	−.0	.2	−.2	−.1	.2	.0	−.0	−.2	.3	−.1	.2	.2	−.0	1		
Ele	.2	4.6	−.5	−.1	.1	.7	−.1	.0	−.0	−.1	.2	−.1	.3	.1	.1	.2	1	
Geo	.5	1.9	.3	.01	−.1	−.0	.2	.2	−.4	.5	−.2	.2	−.3	.0	−.3	−.0	−.1	1

NB. D. Fl (distance to faultline), Rt (rock type), R (rainfall), SoD (soil depth), PrC (profile curvature), PlC (plan curvature); LSF (LS_factor), Sl (slope), Asp (aspect), DR (Distance to rivers), Ele (elevation), Geo (geomorphons).

All variables met acceptable thresholds (VIF < 10; Tol > 0.1; r < 0.9), except for the Terrain Ruggedness Index (TRI), which exhibited high VIF (9.5) and a very strong correlation with slope (r = 0.9). TRI was therefore excluded to avoid redundancy and inflation of model variance. Elevation and rainfall were retained despite showing moderate correlation (r = 0.7), because their VIF (4.6 and 3.5, respectively) and tolerance values indicated no problematic multicollinearity.

#### 2.2.4 Frequency ratio (FR)-maximum entropy (MaxEnt) ensemble modeling.

**Frequency Ratio (FR)**

The FR method is a widely used bivariate GIS techniques for landslides susceptibility assessment in mountainous regions with limited inventory data (32]. The method evaluates the ratio of landslide to non‑landslide occurrences within each class of the conditioning factors [33–35]. In this study, continuous predictors were reclassified into five classes, with Natural Breaks applied to skewed variables and quantile classification to near‑normal ones such as SPI and curvature [83,84]. Aspect, geomorphons, and rock type were not reclassified because they already comprise discrete, implicitly defined classes (Table 3).

**Table 3:** Variable classes, landslide proportions, and frequency ratios.

Factors	Class	Landslide in class	Percentage of Landslide	Area (km^2^)	Percentage of Area	Frequency Ratio	Mean Frequency Ratio
Aspect	Flat	2	3.17	198.22	16.09	0.2	1.00
	North	6	9.52	85.75	6.96	1.37	
	North East	15	23.81	165.52	13.44	1.77	
	East	8	12.70	126.63	10.28	1.24	
	South East	5	7.94	126.05	10.23	0.78	
	South	6	9.52	123.34	10.01	0.95	
	South West	9	14.29	130.01	10.55	1.35	
	West	5	7.94	103.05	8.36	0.95	
	North West	6	9.52	119.36	9.69	0.98	
	North	1	1.59	54.01	4.38	0.36	
Elevation (m)	5–129.99	35	55.56	768.69	62.40	0.89	1.36
	129.99–364.34	15	23.81	277.66	22.54	1.06	
	364.34 −770.55	1	1.59	83.81	6.80	0.23	
	770.55–1,208.01	7	11.11	58.71	4.77	2.33	
	1,208.01–1,997	5	7.94	43.06	3.50	2.27	
Distance from Faultline (m)	0–2,482.82	24	38.10	526.57	42.74	0.89	1.40
	2,482.82–5,763.69	7	11.11	281.06	22.81	0.49	
	5,763.69 −9,931.29	8	12.70	233.95	18.99	0.67	
	9,931.29 −15,340.29	10	15.87	98.56	8.0	1.98	
	15,340.29–22,611.41	14	22.22	91.78	7.45	2.98	
Distance from river	0–169.2	15	23.81	360.75	29.28	0.81	0.87
	169.2–372.24	26	41.27	364.04	29.55	1.4	
	372.24–592.21	15	23.81	284.66	23.11	1.03	
	592.21–888.31	6	9.52	178.02	14.45	0.66	
	888.31–2,157.33	1	1.59	44.46	3.61	0.44	
Geology	Alluvium/mangrove corals/coal corals/conglomerates	19	30.16	549.87	44.63	0.68	052
	Shale/sandstone	1	1.59	53.65	4.35	0.36	
	Andesite/basalt/intrusives	19	30.16	286.99	23.30	1.29	
	Shale/limestone/clay	0	0	0.01	0.00	0	
	Sandstone/shale/siltstone	2	3.17	66.49	5.40	0.59	
	Basalt/andesite/ash	0	0	0.07	0.01	0	
	Interbedded andesite/limestone/ shale	0	0	20.27	1.65	0	
	Granodiorites/Monzonites	0	0	7.47	0.61	0	
	Pyroclast/conglomerates/sandstone/shale	22	34.92	247.11	20.06	1.74	
Geomorphon	Flat	0	0	70.25	5.70	0	0.48
	Peak	0	0	19.73	1.60	0	
	Ridge	3	4.76	103.58	8.41	0.57	
	Shoulder	0	0	14.21	1.15	0	
	Spur	12	19.05	186.47	15.14	1.26	
	Slope	38	60.32	458.03	37.18	1.62	
	Hollow	6	9.52	145.07	11.78	0.81	
	Foot slope	0	0	89.03	7.23	0	
	Valley	4	6.35	141.19	11.46	0.55	
	Pit	0	0	4.35	0.35	0	
LS-factor	0–5.02	24	38.10	883.66	71.73	0.53	0.94
	5.02–17.56	35	55.56	285.89	23.21	2.39	
	17.56–42.65	5	7.94	54.67	4.44	1.79	
	42.65–100.35	0	0	6.9	0.56	0	
	100.35–639.71	0	0	0.82	0.07	0	
NDVI	−0.99 - −0.080	0	0	5.88	0.48	0	1.3
	−0.08. – 0.43	1	1.59	18.57	1.51	1.05	
	0.43–0.69	14	22.22	88.89	7.22	3.08	
	0.69–0.82	29	46.03	295.52	23.99	1.92	
	0.82–0.94	19	30.16	823.07	66.81	0.45	
Plan Curvature	14.35 - - 0.41	17	26.98	239.87	19.47	1.39	1.59
	−0.41 - −0.11	3	4.76	88.74	7.20	0.66	
	−0.11 - −0.01	5	7.94	27.09	2.20	3.61	
	−0.01- 0.58	20	31.75	671.17	54.48	0.58	
	0.58–11.04	18	28.57	205.05	16.64	1.72	
Profile curvature	−17.14 - - 0.62	8	12.70	228.85	18.58	0.68	1.18
	−0.62 - - 0.20	7	11.11	117.91	9.57	1.16	
	− 0.20 - - 0.06	2	3.17	59.61	4.84	0.66	
	−0.06–0.64	27	42.86	684.87	55.59	0.77	
	0.64–18.56	19	30.16	140.69	11.42	2.64	
Rainfall (extreme)	263.85–317.6	16	25.40	391.23	31.76	0.8	0.84
	317.6–349.49	34	53.97	440.49	35.76	1.51	
	349.49–384.11	7	11.11	175.82	14.27	0.78	
	384.11–422.38	4	6.35	161.31	13.09	0.48	
	422.38–496.17	2	3.17	63.08	5.12	0.62	
Soil depth (cm)	0–2,426.18	48	76.19	570.17	46.28	1.65	0.52
	2,426.18–4,270.07	13	20.63	447.16	36.30	0.57	
	4,270.07–9,607.66	2	3.17	101.39	8.23	0.39	
	9,607.66–15,624.58	0	0	31.13	2.53	0	
	15,624.58–24,747.0	0	0	82.08	6.66	0	
SPI	−4.26 - −0.98	3	4.76	240.64	19.53	0.24	1.15
	−0.98 - −0.09	8	12.70	148.53	12.06	1.05	
	−0.09 – - 0.03	1	1.59	11.33	0.92	1.73	
	− 0.03–1.11	35	55.56	641.53	52.08	1.07	
	1.11–11.84	16	25.40	189.9	15.41	1.65	
Slope	0–5.09	4	6.35	532.54	43.23	0.15	1.73
	5.09–12.16	10	15.87	271.72	22.06	0.72	
	12.16–19.80	17	26.98	219.49	17.82	1.51	
	19.80–29.13	22	34.92	151.21	12.27	2.84	
	29.13–72.13	10	15.87	56.97	4.62	3.43	
TWI	1.56–5.43	40	63.49	452.59	36.74	1.73	0.75
	5.43–7.37	17	26.98	442.47	35.92	0.75	
	7.37–9.53	4	6.35	178.72	14.51	0.44	
	9.53–12.58	1	1.59	129.93	10.55	0.15	
	12.58–20.55	1	1.59	28.22	2.29	0.69	

The FR values reported in Table 3 and Supplementary S2 Table for the baseline model and the training-sample allocation scenarios described in Section 2.2.6 were calculated using Equation 5. The Landslide Susceptibility Index (LSI) was subsequently derived by summing the FR values across all conditioning-factor classes, as shown in Equation 6.


$$\begin{array}{c} FR=\frac{Landslide\;points\;in\;class/Total\;landslide\;points}{Class\;area/Total\;study\;area} \end{array}$$ (5)

FR values greater than 1 were interpreted as indicating a positive association between a conditioning‑factor class and landslide occurrence, whereas values below 1 indicated a negative association [85]. The class‑level FR values for each variable were then averaged to obtain a mean frequency ratio per variable (Table 3).


$$\begin{array}{c} LSI=\sum_{i=\text{1}}^{n}FR_{i} \end{array}$$ (6)

where *LSI* denotes the Landslide Susceptibility Index and *FR* represent the frequency ratio for each class of factors.

The LSI raster was normalised to 0–1 using a minimum–maximum transformation and reclassified into five susceptibility classes using the Natural Breaks method. Values approaching 1 were interpreted as high landslide susceptibility, whereas values closer to 0 indicated low susceptibility.

**Maximum entropy (MaxEnt)**

Maximum Entropy (MaxEnt) is a machine‑learning algorithm originally developed for species distribution modelling. Its application has expanded over the past 15 years to include landslide susceptibility assessment [38,39]. It is a presence‑only method that estimates the probability of landslide occurrence from environmental predictors. It does this by identifying the most uniform probability distribution possible, subject to constraints defined by environmental conditions at observed presence locations. The algorithm incorporates a regularisation parameter to limit model complexity, reduce overfitting, and accommodate nonlinear and interactive relationships between landslides and conditioning factors [38]. Conceptually, MaxEnt contrasts the conditional density of predictor variables at presence locations with their marginal (background) density across the study area [37].

This approach is well suited to our presence‑only dataset, as absence data commonly used in landslide susceptibility studies are often pseudo‑absences that may not reliably represent stable conditions [86]. The same set of conditioning factors and landslide inventory were used to train the MaxEnt model in ArcGIS Pro and to validate the outputs in SPSS. Unlike the FR model, which requires binning of conditioning factors, MaxEnt retains continuous predictors in their original form to preserve the full range of environmental variation used to construct linear, quadratic, product, threshold, and hinge features, enabling flexible modelling of nonlinear responses [87,88].

The Maximum Entropy (MaxEnt) model was implemented using linear and product feature expansions to capture both individual effects and pairwise interactions among the conditioning factors. Model calibration was performed within the raster extent of the study area. To minimise spatial autocorrelation among landslide occurrences, spatial thinning was applied using a minimum nearest-neighbour distance of 100 m. The resulting presence observations were assigned a weight of 100 relative to background samples, placing greater emphasis on environmental conditions associated with mapped landslides. Landslide occurrence probability was estimated using the complementary log-log (cloglog) transformation with a probability cutoff of 0.5. To ensure complete susceptibility coverage, predictions were permitted outside the range of the calibration data. In addition to the partitioned landslide inventories used for calibration, 122,641 background samples from the study area were used by the software to characterise the environmental domain against which presence locations were contrasted. The resulting MaxEnt‑derived susceptibility raster was reclassified into five susceptibility classes using the Natural Breaks (Jenks) method, consistent with the classification applied to the FR‑based susceptibility map.

**Ensemble model**

To improve predictive performance, reduce model‑specific bias, and generate a more robust susceptibility map, we constructed an AUC‑weighted ensemble from the FR and MaxEnt models. Prediction‑rate AUCs, which more accurately reflect generalisation ability, were calculated and applied as performance‑based weights, allowing the model with higher predictive skill to contribute proportionally more to the final ensemble output (Equation 7).


$$\begin{array}{c} S_{ensemble}=w_{FR}\text{\textasciitilde{}}x\text{\textasciitilde{}}S_{FR}+w_{MaxEnt}\text{\textasciitilde{}}x\text{\textasciitilde{}}S_{MaxEnt} \end{array}$$ (7)

The equation expresses the ensemble susceptibility value ($S_{\text{ensemble}}$) as a weighted linear combination of the susceptibility outputs from the FR model ($S_{\text{FR}}$) and the MaxEnt model ($S_{\text{MaxEnt}}$). The weights $w_{\text{FR}}$ and $w_{\text{MaxEnt}}$ represent the normalised prediction‑rate AUC values of each model. Because the weights sum to 1, the ensemble value at each pixel is a performance‑based average of the two models. The weight assigned to each model ($w_{i}$) was calculated using Equation 8:


$$\begin{array}{c} w_{i}=\frac{{\text{AUC}}_{i,\text{pred}}}{{\text{AUC}}_{\text{FR},\text{pred}}+{\text{AUC}}_{\text{MaxEnt},\text{pred}}} \end{array}$$ (8)

Using the prediction‑rate AUCs obtained in this study, the FR and MaxEnt models received weights of 0.49 and 0.51, respectively. These weights reflected the slightly higher predictive skill of MaxEnt and ensured that both models contributed meaningfully to the final susceptibility surface. Performance‑based weighting (e.g., AUC‑derived weights) has been shown to reduce model‑specific bias and improve spatial robustness compared with equal weighting, which assumes identical predictive skill [89].

The susceptibility maps were classified using the Natural Breaks (Jenks) method. This approach defines class boundaries by minimizing within-class variance and maximizing between-class variance, thereby preserving the inherent distribution of continuous susceptibility values [90]. Because landslide susceptibility data are typically non-uniform and spatially clustered, the Jenks method provides a more realistic representation of hazard zones than equal-interval or quantile classifications, which rely on arbitrary or percentile-based thresholds [91]. Consequently, it enhances the distinction between low- and high-susceptibility areas while better capturing natural variations in susceptibility across the landscape.

#### 2.2.5 Model validation.

Model validation is a critical component of landslide susceptibility assessment [85,92]. The performance of the FR, MaxEnt, and ensemble models was evaluated using two complementary approaches: (i) the distribution of mapped landslides across susceptibility classes and (ii) receiver operating characteristic-area under the curve (ROC-AUC) analyses based on success-rate and prediction-rate curves. The susceptibility-class analysis quantified the proportion of landslides occurring within each susceptibility class, providing an intuitive measure of how effectively each model concentrated landslides within highly susceptible zones.

ROC-AUC analysis was used to assess the ability of each model to discriminate between landslide and non-landslide conditions, with uncertainty quantified using 95% confidence intervals. An additional 10,000 randomly generated background points were used to represent the environmental variability of the study area and provide a reference environmental domain for comparison with landslide locations. A binary response field was created by assigning a value of 1 to landslide locations and 0 to background points. Susceptibility values from the FR, MaxEnt, and ensemble rasters were extracted to all points using the Extract Multi Values to Points tool in ArcGIS Pro. Success-rate ROC-AUCs were calculated using training landslides and background points, whereas prediction-rate ROC-AUCs were calculated using the withheld validation landslides and the same background dataset in SPSS version 20. Because prediction-rate AUCs provide a more rigorous assessment of model generalisation, they were used to derive the performance-based weights applied in the ensemble model.

#### 2.2.6 Sensitivity analysis of training-validation allocation.

To assess the influence of inventory allocation on model performance, a sensitivity analysis was conducted using 65:35% and 80:20% training-validation partitions in addition to the 72.4:27.6% baseline model. These partitions corresponded to 57 and 70 calibration landslide occurrences and 30 and 17 validation occurrences, respectively. For each alternative partition, the FR and MaxEnt models were recalibrated using identical predictor variables and model settings, and the FR-MaxEnt ensemble was regenerated using the same AUC-weighting procedure (Equation 8). Model sensitivity was evaluated by comparing success-rate and prediction-rate ROC curves, corresponding AUC values, and the distribution of mapped landslides across susceptibility classes. The alternative partitions were used exclusively for sensitivity assessment and did not replace the baseline model used for susceptibility mapping, hotspot analysis, and road-exposure evaluation. Model performance under these partitions was compared directly with that of the baseline model to assess the influence of inventory allocation.

#### 2.2.7 Hot spot and road exposure analysis.

Hotspot analysis is an important yet often overlooked component of susceptibility assessment [86,93,94]. To identify statistically significant clusters of high and low susceptibility, we applied the Getis‑Ord Gi* statistic in ArcGIS Pro using vectorised ensemble‑susceptibility points. The Gi* statistic evaluates each pixel relative to its surrounding neighbourhood to determine whether it forms part of a cluster of unusually high (hot spot) or low (cold spot) values. Spatial relationships were defined using a fixed‑distance band of 12.5 m, matching the resolution of the ensemble raster to capture the smallest meaningful neighbourhood and preserve micro‑scale clustering patterns. Larger neighbourhoods (25 m and 50 m) were tested but excluded because they produced generalised patterns unsuitable for corridor‑scale planning. The final analysis identified statistically significant hot and cold spots at the 90%, 95%, and 99% confidence levels, supporting the identification of priority intervention zones along the road network.

To quantify road exposure, the regional road network was overlaid on the ensemble susceptibility map. The Summary Statistics tool in ArcGIS Pro was used to calculate the length of primary roads intersecting high‑susceptibility zones. The analysis focused on primary roads—including the interprovincial corridor to David and the Changuinola–Guabito–Las Delicias corridor—because secondary and tertiary roads lacked consistent naming in the OpenStreetMap dataset.

#### 2.2.8 Variable importance.

Variable importance was assessed for the FR, MaxEnt, and ensemble models. For the FR model, importance was derived by averaging frequency‑ratio values across predictor classes (Table 3), following established approaches in landslide‑susceptibility research [31,35].

For MaxEnt, we used permutation importance, a path‑independent and statistically robust metric widely recommended over percent contribution for reliable variable ranking [37,95]. Variable explanatory power was further assessed using the Jackknife test in the standalone MaxEnt software (version 3.4.4), which evaluates model performance when predictors are excluded or used individually. Although MaxEnt was executed in ArcGIS Pro, variable‑importance analysis was performed in the standalone software because ArcGIS Pro does not generate permutation‑importance outputs.

The ensemble‑model variable importance was derived by computing a weighted average of the FR mean values and MaxEnt permutation‑importance scores for each predictor, using the same AUC‑based weights applied in the ensemble formulation. The resulting values were normalised to sum to 100%, yielding a clear and interpretable ranking of each variable’s integrated influence within the ensemble. The Jackknife test provided complementary evidence, reinforcing the robustness and consistency of variable‑influence patterns across modelling frameworks.

## 3 Results

### 3.1 Landslide susceptibility patterns

The FR, MaxEnt, and AUC‑weighted ensemble models produced broadly consistent spatial patterns of landslide susceptibility along the road corridor (Fig 7a–7c). These patterns closely aligned with the statistically significant hot and cold spots identified through the Getis‑Ord Gi* analysis (Fig 7d).

**Fig 7:**
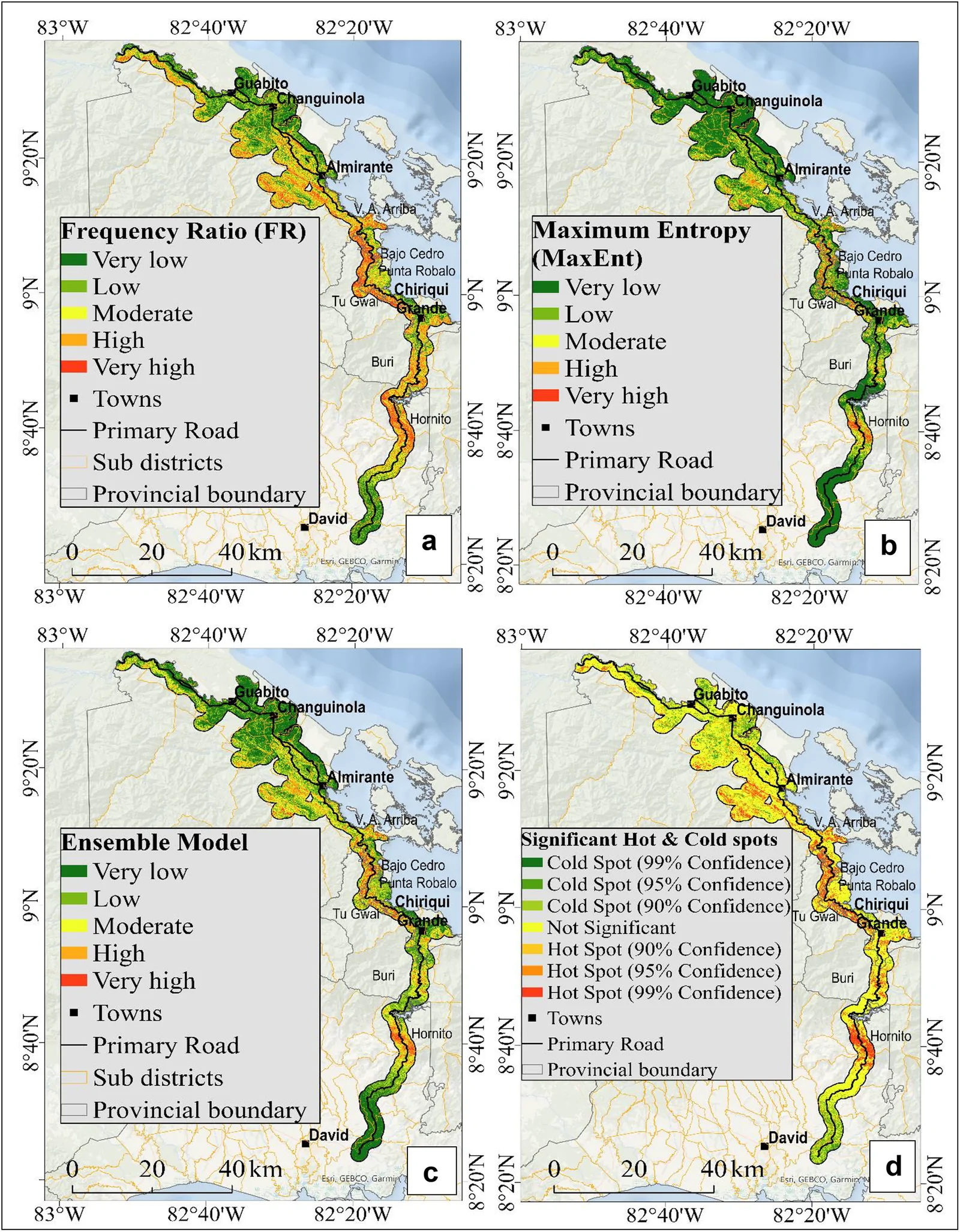
Landslide susceptibility maps from Frequency Ratio, Maximum Entropy, Ensemble models, with statistically significant hotspots NB. V.A. Arriba is Valle de Agua Arriba.

The FR model (Fig 7a) delineated relatively extensive high-susceptibility zones, reflecting the strong influence of the regional topographic gradient (Fig 4b). High and very high susceptibility classes together occupied 29.92% of the corridor, whereas low and very low classes accounted for 46.64% (Table 4). In contrast, the MaxEnt model (Fig 7b) produced a more conservative susceptibility pattern, with only 5.46% and 3.03% of the corridor classified as high and very high susceptibility, respectively. These zones were concentrated in steep and rugged terrain, particularly around Hornito, Buri, Bajo Cedro, and Valle de Agua Arriba, while more than 80% of the corridor was classified as low or very low susceptibility. The contrasting susceptibility patterns reflect differences in model structure, with FR capturing bivariate relationships between landslide occurrence and individual conditioning factors, whereas MaxEnt accounts for nonlinear interactions among multiple predictors.

**Table 4:** Absolute and relative proportion of area under each susceptibility class.

Susceptibility	FR Model		MaxEnt Model		AUC Ensemble Model	
	Area (km^2^)	%	Area (km^2^)	%	Area (km^2^)	%
Very low	256.86	20.85	698.26	56.68	434.02	35.23
Low	317.74	25.79	287.38	23.33	377.02	30.60
Moderate	291.12	23.63	141.81	11.51	252.59	20.50
High	237.3	19.26	67.21	5.46	119.18	9.67
Very high	128.92	10.46	37.27	3.03	49.12	3.99
**TOTAL**	**1231.93**	**100**	**1231.93**	**100**	**1231.93**	**100**

The ensemble model (Fig 7c) generated a more spatially coherent susceptibility surface, with 13.66% of the area mapped as high and very high susceptibility and the remainder distributed across moderate (20.50%), low (30.60%), and very‑low (35.23%) classes. The ensemble approach reduced isolated high‑susceptibility pixels and strengthened areas where FR and MaxEnt converged, consistent with the stabilising effect of model averaging.

Pearson correlations between susceptibility outputs and conditioning factors (Fig 8) showed strong positive associations with slope and moderate positive correlations with SPI, LS‑factor, and elevation. TWI exhibited a strong negative correlation.

**Fig 8:**
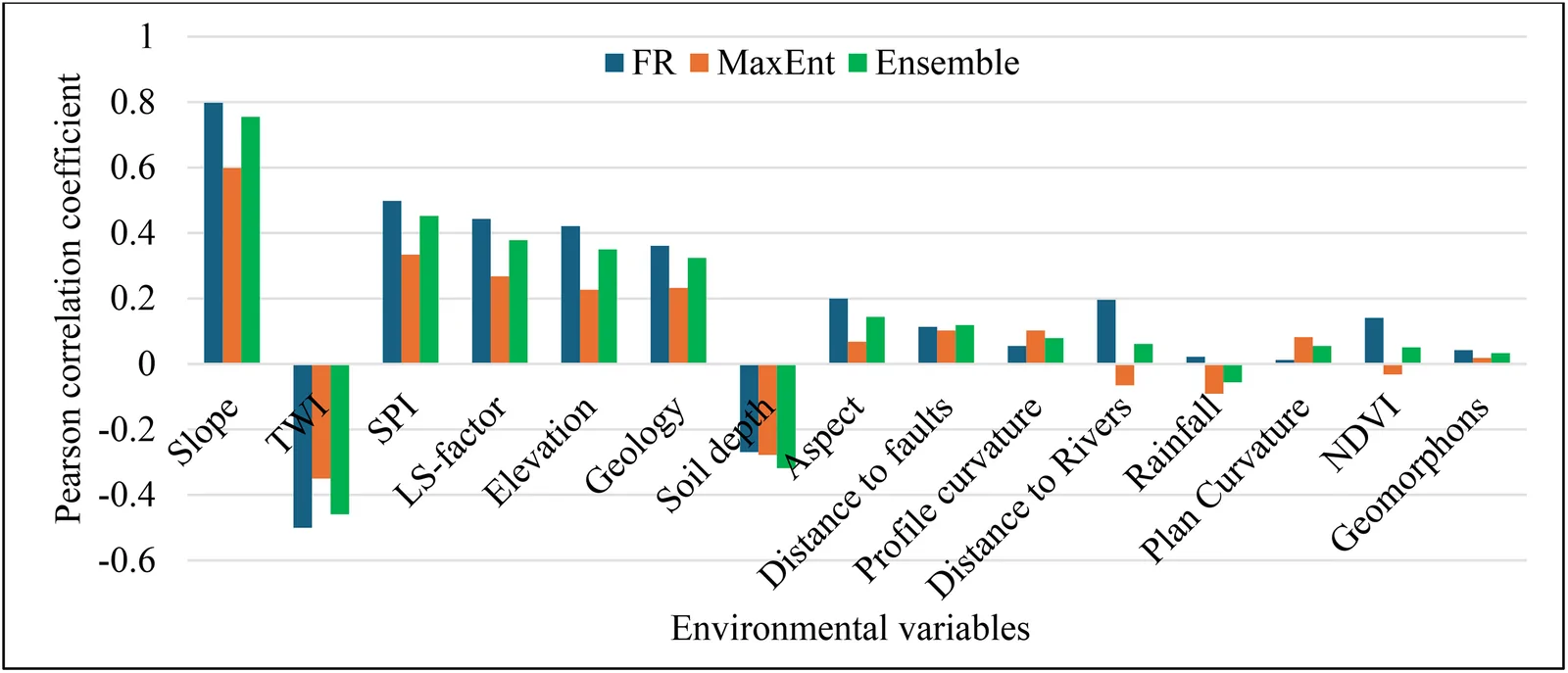
Pearson correlation across the models for landslide susceptibility and environmental variables.

These relationships suggest that steep slopes, concentrated runoff, and shallow soils are key drivers of susceptibility. NDVI showed a weak positive correlation, reflecting the tendency for intermediate vegetation density to coincide with steep, moisture‑rich slopes. Curvature metrics and rainfall showed weak correlations, suggesting limited alignment between their class distributions and susceptibility patterns.

### 3.2 Statistically significant landslide hot spots

Hotspot analysis of the ensemble raster identified statistically significant clusters of high and low susceptibility at the 90%, 95%, and 99% confidence levels (Fig 7d). High‑confidence hotspots (95% and 99%) closely aligned with the high‑susceptibility zones delineated by the MaxEnt and ensemble models, whereas cold spots were confined to the flat, low‑susceptibility terrain around Changuinola, Chiriquí Grande, and David. More than 65% of the corridor was classified as “not significant,” indicating that susceptibility values did not form coherent spatial clusters and that isolated high or low‑value pixels lacked neighbourhood reinforcement. These patterns collectively highlight spatially reinforced zones where slope failures are most likely to cluster, providing actionable guidance for corridor‑scale planning.

Although detailed road-disruption records were unavailable at a spatial resolution suitable for formal statistical evaluation, Fig 7d shows that most high-confidence hotspot clusters coincide with primary road segments where recurrent slope instabilities were recorded during field surveys. This correspondence suggests that the hotspot analysis identified statistically significant concentrations of elevated susceptibility and highlighted road sections repeatedly affected by slope failures.

### 3.3 Model performance and validation

#### 3.3.1 Landslide frequency distribution.

All three models concentrated most landslides within the upper susceptibility classes, but with notable differences (Table 5).

**Table 5:** Relative proportions of observed landslides within each model’s susceptibility class.

Susceptibility	FR Model			MaxEnt Model			AUC Ensemble Model		
	Area (km^2^)	F	%	Area (km^2^)	F	%	Area (km^2^)	F	%
Very low	256.86	1	1.15	698.26	2	2.30	434.02	1	1.15
Low	317.74	0	0.0	287.38	8	9.20	377.02	2	2.30
Moderate	291.12	8	9.20	141.81	15	17.24	252.59	13	14.94
High	237.3	26	29.89	67.21	18	20.69	119.18	22	25.29
Very high	128.92	52	59.77	37.27	44	50.57	49.12	49	56.32
**TOTAL**	**1231.93**	**87**	**100**	**1231.93**	**87**	**100**	**1231.93**	**87**	**100**

NB. F = frequency of landslide points in each susceptibility class.

Although 89.66% of mapped landslides in the FR model occurred within the high and very high susceptibility classes, these classes occupied 366.22 km^2^ of the study area, indicating a tendency towards spatial overprediction. In contrast, 71.26% of landslides in the MaxEnt model occurred within the two highest susceptibility classes, while 17.24% remained in the Moderate class, reflecting a more diffuse susceptibility pattern. The FR-MaxEnt ensemble provided a more balanced representation, capturing 81.61% of landslides within the high and very high classes, with only 3.45% occurring in the low and very low classes compared with 11.50% in the MaxEnt model. The ensemble therefore achieved a favourable balance between concentrating landslides within highly susceptible zones and limiting the spatial overgeneralisation observed in the FR model.

#### 3.3.2 Receiver operating characteristics (ROC)-area under the curve (AUC) curves.

ROC–AUC curves (Fig 9) and confidence intervals (Table 6) reveal clear differences in model performance.

**Table 6:** Standard error and confident intervals of the models’ performance.

Model	Success Rate		Prediction Rate	
	Std. Error	95% Confident Interval(Lower–Upper)	Std. Error	95% Confident Interval(Lower–Upper)
FR	0.020	0.835–0.915	0.020	0.833–0.913
MaxEnt	0.019	0.867–0.941	0.014	0.916–0.970
Ensemble	0.017	0.879–0.947	0.013	0.920–0.969

**Fig 9:**
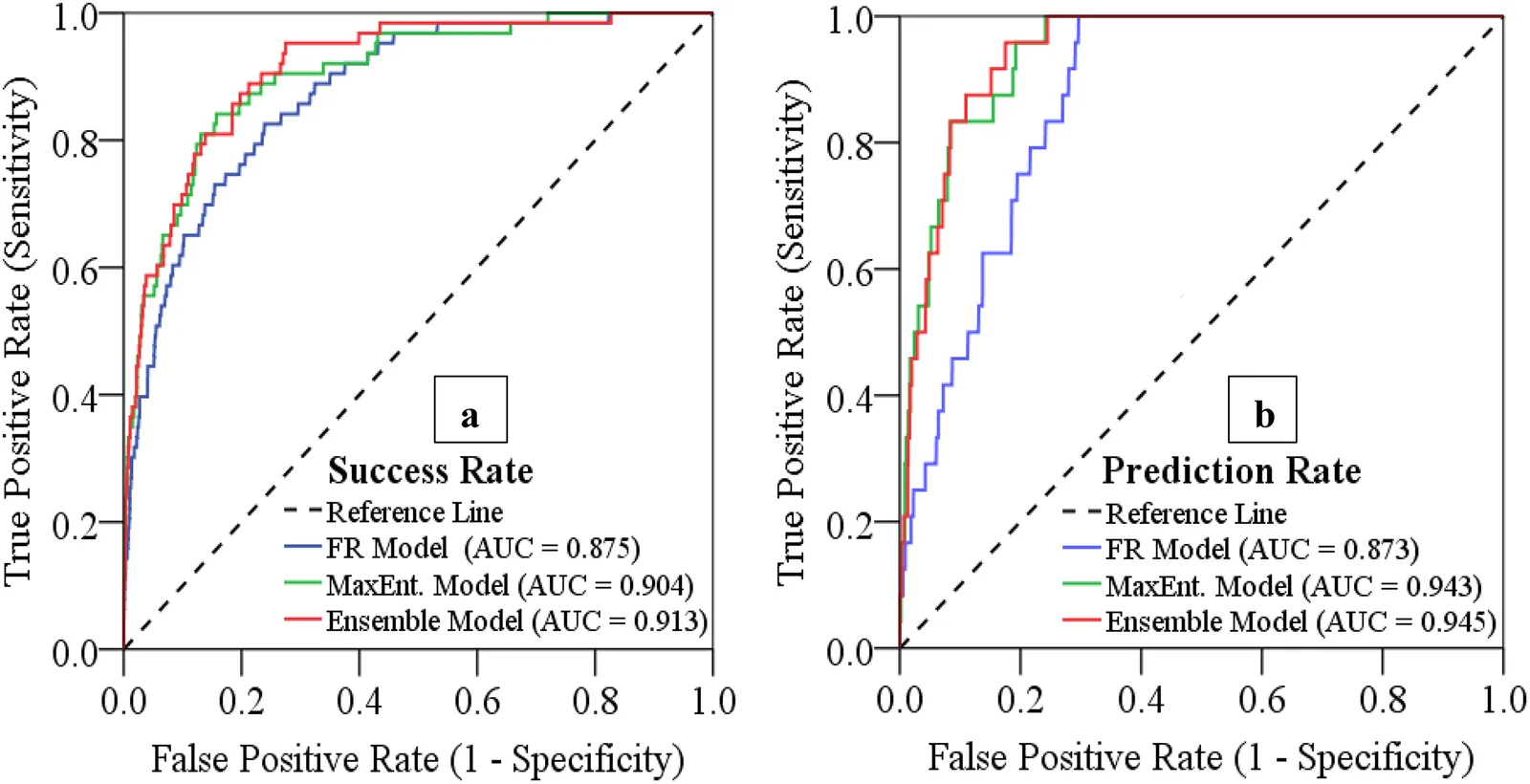
ROC success and prediction rate curves for FR, MaxEnt, and Ensemble models. NB. All AUC values are significant at p < 0.001 under the non-parametric assumption.

The FR model achieved success-rate and prediction-rate AUC values of 0.875 and 0.873, respectively. MaxEnt performed better, with corresponding AUC values of 0.904 and 0.943, while the FR-MaxEnt ensemble attained the highest values (0.913 and 0.945). The ensemble also exhibited the lowest standard error and narrowest confidence interval among the baseline models (Table 6), further supporting its reliability. This improvement reflects the complementary strengths of the two constituent models, with FR providing interpretable factor-response relationships and MaxEnt capturing nonlinear interactions among multiple conditioning factors.

#### 3.3.3 Model performance under alternative training-validation partitions.

Under the 65:35% partition, the FR, MaxEnt, and FR-MaxEnt ensemble models achieved success-rate AUCs of 0.913, 0.923, and 0.932, respectively, and prediction-rate AUCs of 0.835, 0.875, and 0.881. Under the 80:20% partition, the corresponding success-rate AUCs were 0.898, 0.915, and 0.925, whereas prediction-rate AUCs were 0.852, 0.909, and 0.902 (S1 Fig). Relative to the baseline model, the alternative partitions produced slightly higher success-rate AUCs but generally lower prediction-rate AUCs. Nevertheless, all prediction-rate AUC values exceeded 0.80, indicating consistently good discrimination across the tested inventory allocations.

For the ensemble model, both alternative partitions yielded a standard error of 0.029, with 95% confidence intervals of 0.824–0.937 and 0.845–0.959 for the 65:35% and 80:20% partitions, respectively (S3 Table). Susceptibility-class statistics for the ensemble model were broadly consistent across the baseline and alternative inventory allocations (S4 Table). The high and very high susceptibility classes contained 82.76%, 81.61%, and 81.61% of mapped landslides under the 65:35%, 80:20% (S2-S3 Fig; S4 Table), and baseline partitions (Table 5), respectively, while occupying 186.67, 178.98, and 168.30 km^2^. Although the FR model assigned more than 90% of mapped landslides to the high and very high classes in both sensitivity scenarios, these classes occupied more than 400 km^2^, indicating lower spatial selectivity. In contrast, the ensemble consistently concentrated more than four-fifths of mapped landslides within approximately 14–15% of the study area across all inventory allocations. Together with the consistently high prediction-rate AUC values, these findings suggest that the relative performance of the tested models and the general susceptibility patterns were only moderately influenced by the alternative inventory allocations considered.

### 3.4 Exposure of road network to landslide

This quantified road‑exposure analysis provides a practical basis for prioritising infrastructure management under future climate variability. Of the 266.55 km of primary roads in the study area, 71.67 km intersected high and very‑high susceptibility zones. Fig 10 illustrates the spatial distribution of exposure across subdistricts, from Gualaca (left) near the city of David to Las Delicias (right) in the district of Changuinola.

**Fig 10:**
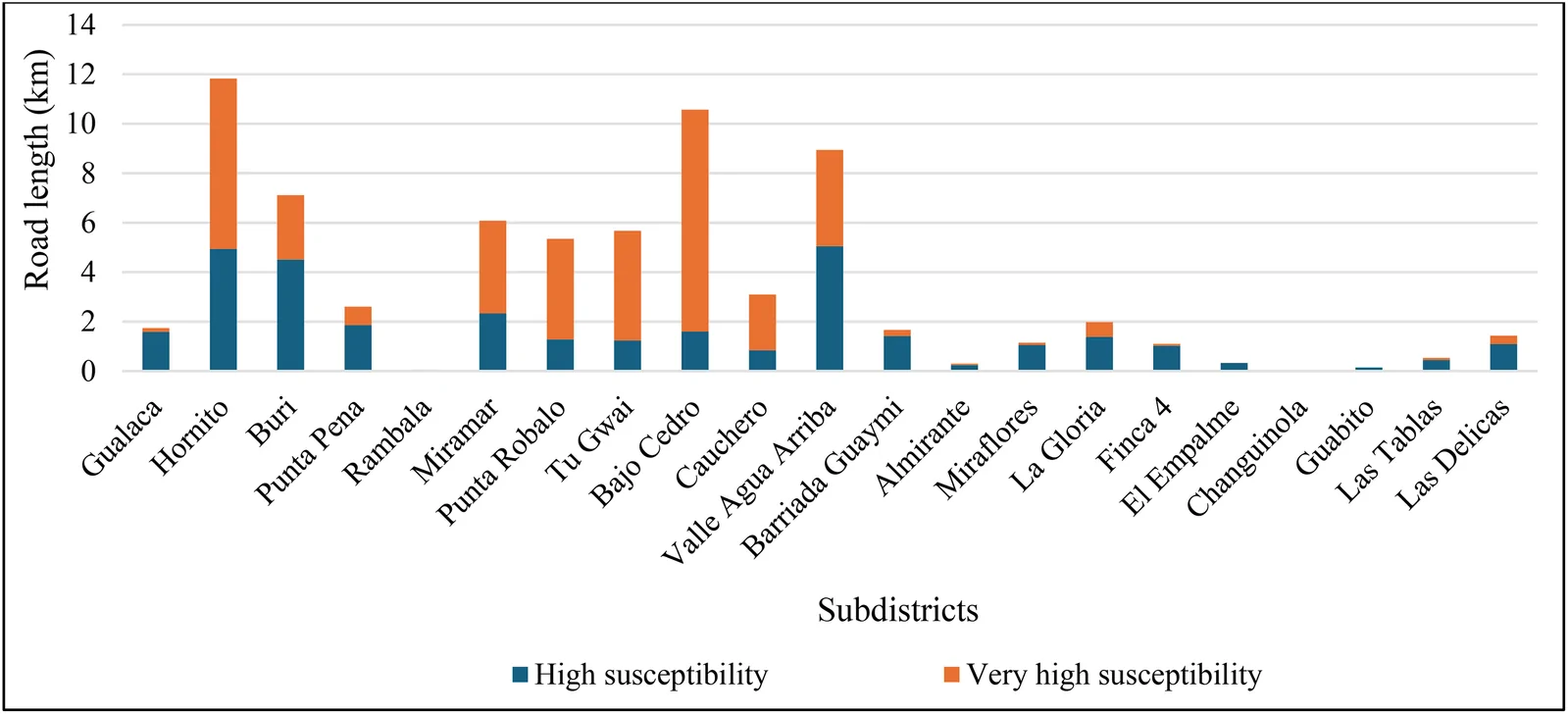
Lengths of primary roads intersecting highly susceptible zones across subdistricts.

The most exposed subdistricts—Hornito (11.83 km), Bajo Cedro (10.57 km), and Valle de Agua Arriba (8.94 km)—correspond directly to the 99%‑confidence hotspot clusters, indicating that these segments represent the highest‑priority corridors for intervention. The subdistricts of Buri, Miramar, Punta Robalo, and Tu Gwai, with more than 5 km of exposed roads also align with statistically significant hotspots. Together, these hotspot‑aligned segments represent priority corridors where slope failures are most likely to cluster and where cost‑effective mitigation efforts can be concentrated.

High‑confidence hotspots should be targeted for enhanced monitoring of rainfall‑triggered landslide hazards. Engineering‑based stabilisation measures such as drainage improvements and reinforcement of cut‑slopes should be applied in the most exposed sections. Ecosystem‑based mitigation, including vegetation restoration and bio‑engineering, should be implemented in adjacent moderate‑susceptibility zones to reduce long‑term vulnerability under projected increases in rainfall intensity.

### 3.5 Variable importance

Variable‑importance analysis (Fig 11a) showed that NDVI and slope are the dominant predictors in the ensemble model, reflecting their strong influence in both the FR and MaxEnt models. The Jackknife results (Fig 11b) further confirmed that NDVI provides unique vegetation‑related information not captured by other predictors, while slope yielded the highest individual gain.

**Fig 11:**
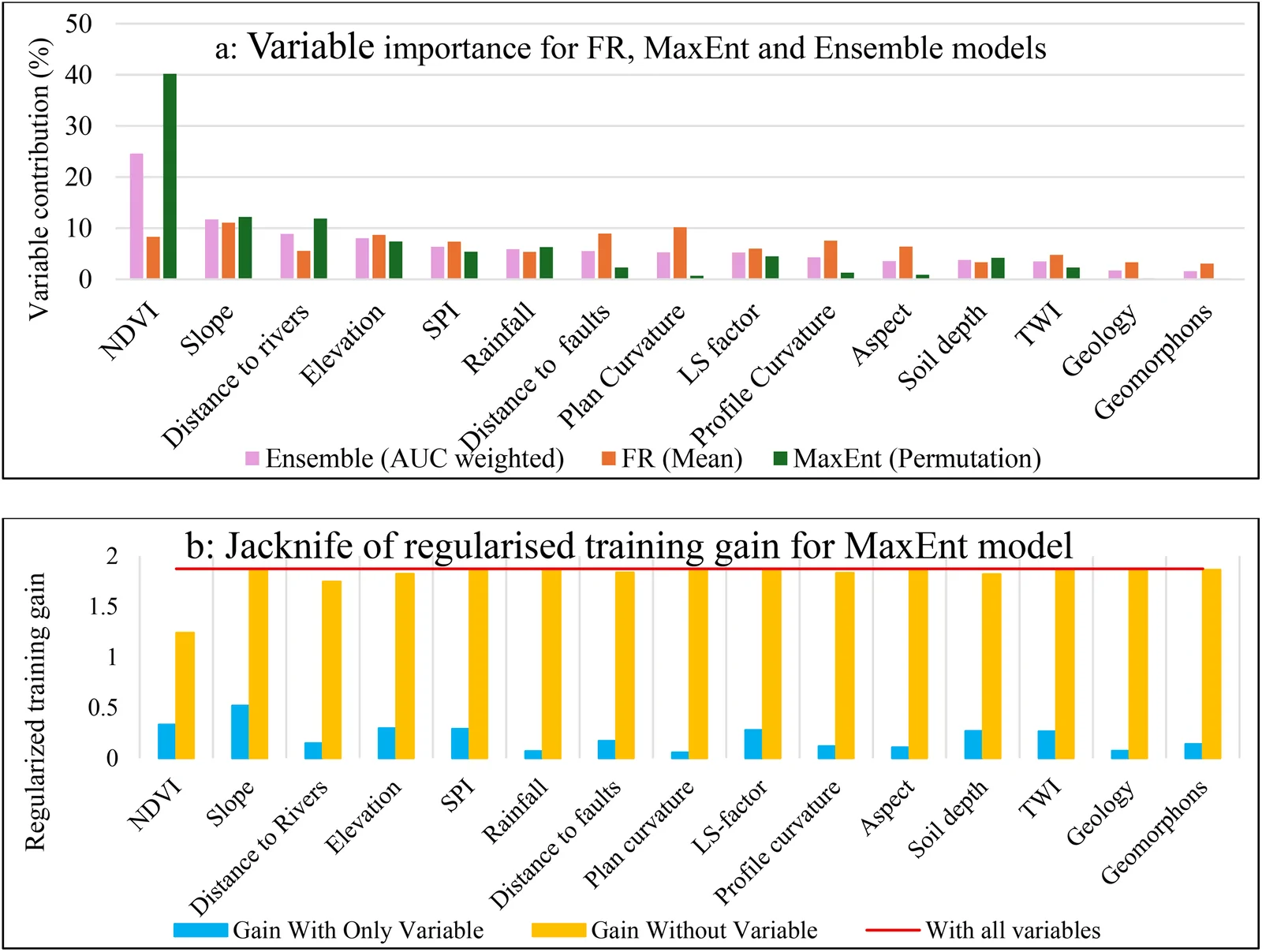
Variable importance and Jacknife training gain for environmental variables.

Distance to rivers, rainfall, and soil depth showed moderate contributions in MaxEnt but lower influence in FR, indicating that MaxEnt captures interactions not represented in bivariate analysis. Curvature metrics and aspect contributed more strongly to FR but had limited influence in MaxEnt, whereas geology and geomorphons exhibited consistently low contributions across all models.

## 4 Discussion

The FR–MaxEnt ensemble model provides a robust and transparent framework for landslide susceptibility assessment along road corridors in western Panama. By combining the interpretable factor–response relationships of FR with the nonlinear, presence‑only strengths of MaxEnt, the ensemble produced more stable and spatially coherent predictions than either model alone. These improvements are consistent with recent ensemble‑based landslide studies, which show that hybrid approaches reduce individual model biases and enhance generalisation capacity [91,96].

This corridor‑focused approach addresses a critical gap in landslide research across Latin America and the Caribbean, where detailed assessments linking hazard patterns to infrastructure exposure remain limited [17,86,94]. Road networks in the region have low density and serve as essential lifelines [27], making fine‑scale susceptibility mapping particularly relevant for climate‑resilient transportation planning. The integration of susceptibility modelling with exposure analysis in this study therefore provides actionable insights for prioritising interventions along critical segments of the network.

Model validation results indicate that predictive discrimination remained strong across the tested inventory allocations, although performance varied with the calibration-validation partition. The baseline model produced the highest prediction-rate AUC values, whereas the alternative partitions yielded slightly higher success-rate AUCs. Nevertheless, the FR-MaxEnt ensemble maintained strong predictive performance across all scenarios and remained comparable to MaxEnt under the 80:20% partition. Together, the consistently high AUC values, low standard errors, and relatively narrow confidence intervals suggest that the principal interpretation was only moderately influenced by the alternative inventory allocations examined. However, because each allocation was evaluated only once and no external inventory was available, these findings should be interpreted as evidence of stability across the tested partitions rather than proof of inventory adequacy or general robustness to repeated random resampling. This interpretation is further supported by the susceptibility-class analysis, which showed that the ensemble consistently concentrated more than 80% of mapped landslides within the high and very high susceptibility classes while avoiding the broader spatial allocation of highly susceptible terrain observed in the FR model (Table 5; S4 Table). These findings are consistent with previous studies highlighting the strong predictive capability of MaxEnt in presence-only susceptibility modelling [37,38,95] and the interpretability of FR for susceptibility assessment and decision-making [32,35].

Spatial coherence between susceptibility outputs and hotspot clusters provides additional support for the ensemble model. Hotspot analysis identifies statistically significant concentrations of elevated hazard rather than isolated high‑value pixels [86,93]. The close alignment between 95–99% confidence hotspots and high‑susceptibility zones indicate that the mapped high‑risk areas represent meaningful spatial clusters of landslide‑prone terrain. This convergence is particularly important for corridor‑scale planning, where interventions must be prioritised along linear infrastructure networks.

Variable‑importance results highlight slope and NDVI as dominant predictors, consistent with global evidence that steep terrain and vegetation structure strongly influence landslide occurrence [30,79,97]. The MaxEnt Jackknife analysis confirmed that NDVI provides unique explanatory power, reflecting the complex role of vegetation in tropical environments where biomass can both stabilise soils and increase gravitational loading [98]. Hydrological variables such as SPI and LS‑factor also contributed meaningfully, supporting previous findings that concentrated runoff and slope‑length drive shallow failures [75,76]. The relatively weak influence of long‑term rainfall aligns with studies showing that rainfall acts primarily as a temporal trigger rather than a spatial determinant of susceptibility [48,99].

The exposure analysis underscores the practical relevance of the modelling framework. More than 71 km of primary roads intersect high‑susceptibility zones, with the highest exposure in Hornito, Bajo Cedro, and Valle de Agua Arriba subdistricts that correspond closely to the 99%‑confidence hotspots. These segments therefore represent priority locations for monitoring, slope stabilisation, and drainage improvements. Similar corridor‑scale studies emphasise the importance of integrating susceptibility outputs with infrastructure exposure to guide targeted interventions [19,49]. The FR–MaxEnt ensemble thus offers a practical, reproducible, and scalable tool for supporting climate‑resilient transportation planning in western Panama and comparable tropical environments.

Recent methodological advances further support the scientific basis of this approach. Multi‑factor frameworks have demonstrated strong performance in mountainous and tectonically active regions, with geomorphological and vegetation controls emerging as dominant predictors [100]. Infrastructure‑focused studies similarly highlight the vulnerability of road corridors in hazard‑prone environments and the need for targeted identification of critical segments [101,102]. Advances in fuzzy logic, machine‑learning classifiers, factor‑screening techniques, and information‑value‑based ensemble models continue to improve predictive reliability in data‑limited settings [97,103,104]. Collectively, these studies reinforce the value of ensemble modelling for strengthening hazard preparedness along critical transportation networks in western Panama.

### 4.1 Limitations and future work

Despite its strengths, this study has several limitations. The landslide inventory of 87 mapped events, although valuable, is relatively small and constrains statistical robustness. Expanding the inventory through field surveys or remote‑sensing interpretation would improve model calibration and validation. Rainfall was represented using long‑term 90th‑percentile values, which capture broad climatic gradients but not the event‑specific triggers that typically initiate slope failures. Incorporating dynamic rainfall metrics—such as intensity–duration thresholds or antecedent rainfall indices—would enhance temporal realism, particularly under changing climatic conditions.

Several conditioning datasets, notably soil depth (250 m) and rainfall (1 km), were coarse relative to the fine‑scale geomorphology of road corridors, introducing spatial uncertainty that may obscure local terrain controls. Higher‑resolution soil and rainfall products would strengthen fine‑scale susceptibility estimates. In addition, the study area was defined using a 1,000 m buffer along road corridors. Evaluating alternative buffer widths would help assess the sensitivity of susceptibility patterns to this spatial assumption.

Future work should therefore integrate larger and more detailed inventories, event‑based rainfall metrics, climate‑change scenarios, higher‑resolution environmental datasets, and buffer‑sensitivity analyses to refine local‑scale susceptibility assessments.

## 5 Conclusion

This study demonstrates the utility of an AUC-weighted FR–MaxEnt ensemble for landslide susceptibility assessment along road corridors in data-limited tropical mountain environments. By combining the interpretability of FR with the nonlinear predictive capability of MaxEnt, the ensemble generally improved discrimination relative to the individual models and produced susceptibility patterns consistent with observed landslide concentrations and statistically significant hotspot clusters. It also concentrated a high proportion of mapped landslides within a smaller high and very high susceptibility area than the FR model, providing a more spatially selective basis for identifying priority zones. The susceptibility maps identified road sections in Hornito, Bajo Cedro, and Valle de Agua Arriba where slope failures are most likely to disrupt transportation networks and where monitoring, slope stabilisation, drainage improvement, and ecosystem-based interventions could be prioritised. Performance remained strong under the two alternative calibration–validation partitions, and similar ensemble class-frequency and area patterns indicated that the principal susceptibility interpretation was not strongly affected by the specific inventory allocations tested. These findings support use of the baseline ensemble for the principal analysis but do not establish robustness across a broader range of sample allocations. Repeated partitioning, cross-validation, and validation with an external landslide inventory would provide a stronger assessment. Future work should also incorporate larger inventories, event-based rainfall information, higher-resolution terrain and soil datasets, climate-change scenarios, and dynamic rainfall thresholds. Overall, the FR–MaxEnt ensemble provides a practical and reproducible framework for risk-informed transportation planning in western Panama and comparable data-limited tropical regions.

## Supporting information

S1 Table Georeferenced landslide points for “Ensemble Frequency Ratio – Maximum Entropy Modelling of Landslide Susceptibility for Climate‑Resilient Road Corridors in Bocas del Toro and Chiriqui, Panama”. (DOCX)

S2 Table Frequency ratios for the 65% and 80% training partitions. (DOCX)

S3 Table AUCs Standard errors and confident intervals for the 65% and 80% sensitivity scenarios. (DOCX)

S4 Table Relative proportions of observed landslides within each model’s susceptibility class for the sensitivity scenarios. (DOCX)

S1 Fig ROC-AUCs success and prediction rates for the 65:35% and 80:20% inventory allocation scenarios. (DOCX)

S2 Fig Frequency ratio, maximum entropy and ensemble landslide susceptibility patterns for the 65% training partition scenario. (DOCX)

S3 Fig Frequency ratio, maximum entropy and ensemble susceptibility patterns for the 80% training partition scenario. (DOCX)

## Abbreviations

ALOS: Advanced Land Observing Satellite
AUC: Area Under the Curve
DEM: Digital Elevation Model
FR: Frequency ratio
GIS: Geographic Information System
GPS: Global Positioning System
LS factor: Slope length and steepness factor
LSI: Landslide Susceptibility Index
MaxEnt: Maximum Entropy
NDVI: Normalize Different Vegetation Index
PALSAR: Phased Array type L‑band Synthetic Aperture Radar
ROC: Receiver Operating Characteristics
RTC: Radiometric Terrain Corrected
RUSLE: Revised Universal Soil Loss Equation
SPI: Stream Power Index
SPSS: Statistical Package for Social Sciences
Tol: Tolerance
TRI: Terrain Ruggedness Index
TWI: Topographic Wetness Index
UTM: Universal Transverse Mercato
VIF: Variance Inflation Factor
WGS: World Geodetic System
