# Supplementary material for: Ensemble frequency ratio-maximum entropy landslide susceptibility modelling for climate‑resilient road corridors in Bocas del Toro and Chiriquí, Panama
Source: PLoS One. 2026 Nov 5;21(11):e0362084. doi: 10.1371/journal.pone.0362084 (PMC13739119; doi:10.1371/journal.pone.0362084)
Supplement: Supplementary file 1 [file pone.0362084.s001.docx]

**Georeferenced landslide points for “Ensemble Frequency Ratio – Maximum Entropy Modelling of Landslide Susceptibility for Climate‑Resilient Road Corridors in Bocas del Toro and Chiriqui, Panama”**

Dereck Mbeh Petiangma^1a^, Gerald G. Singh^2^, Enrique F. Williams^3^, Jeffrey Gerardo Ramos Perez^4^, Rachel Collin^1b^

*^1^Smithsonian Tropical Research Institute, Bocas Research Station, Bocas del Toro, Panama*

*^2^School of Environmental Studies, University of British Columbia, Canada*

*^3^Universidad de Panama, Centro Regional de Bocas del Toro, Panama*

*^4^Universidad Tecnologica de Panama, Centro Regional de Bocas del Toro, Panama*

*1a Current address: Disaster Management Training and Education Center for Africa (DiMTEC), University of the Free State, Bloemfontein, South Africa*

***Corresponding Author:** Dereck Mbeh Petiangma	**Email:** pdmbeh23@hotmail.com

**1. Introduction**

This supplementary material provides the geographic coordinates of the 87 landslide points mapped along road corridors in Bocas del Toro Province, including those along the interprovincial highway connecting the province to the rest of Panama (Table 1).

**Landslide inventory**

Table 1 presents the geographic coordinates of the 87 landslide scars mapped with a Garmin GPS unit along road corridors in the study area. The inventory was randomly divided into 72.4% (63 scars) for model training and 27.6% (24 scars) for validation

S1 Table: Georeferenced Landslide points along road corridors in Bocas del Toro and Chiriqui, Panama

| SN | Latitude | Longitude |
| --- | --- | --- |
| 1 | 9.23194 | -82.412 |
| 2 | 9.23107 | -82.4117 |
| 3 | 9.23106 | -82.4117 |
| 4 | 9.2309 | -82.4117 |
| 5 | 9.23216 | -82.4266 |
| 6 | 9.22253 | -82.4313 |
| 7 | 9.25475 | -82.4806 |
| 8 | 9.25416 | -82.4865 |
| 9 | 9.2525 | -82.4882 |
| 10 | 9.24005 | -82.4956 |
| 11 | 9.23397 | -82.5015 |
| 12 | 9.3413 | -82.4759 |
| 13 | 9.34149 | -82.4764 |
| 14 | 9.33848 | -82.4783 |
| 15 | 9.35409 | -82.577 |
| 16 | 9.35556 | -82.5789 |
| 17 | 9.23393 | -82.3818 |
| 18 | 9.16703 | -82.3148 |
| 19 | 9.16345 | -82.313 |
| 20 | 9.14799 | -82.3236 |
| 21 | 9.14867 | -82.3153 |
| 22 | 9.15071 | -82.3109 |
| 23 | 9.14842 | -82.3065 |
| 24 | 9.14799 | -82.3055 |
| 25 | 9.12278 | -82.2988 |
| 26 | 9.12157 | -82.2981 |
| 27 | 9.08728 | -82.289 |
| 28 | 9.08703 | -82.2886 |
| 29 | 9.0864 | -82.2882 |
| 30 | 9.08237 | -82.2855 |
| 31 | 9.06703 | -82.299 |
| 32 | 9.04745 | -82.3024 |
| 33 | 9.03593 | -82.3032 |
| 34 | 9.0227 | -82.2884 |
| 35 | 9.01928 | -82.2825 |
| 36 | 9.01679 | -82.2822 |
| 37 | 9.01428 | -82.2746 |
| 38 | 8.95832 | -82.199 |
| 39 | 8.9631 | -82.2021 |
| 40 | 8.975 | -82.2114 |
| 41 | 9.07868 | -82.2886 |
| 42 | 9.07824 | -82.2886 |
| 43 | 9.270489 | -82.4109 |
| 44 | 9.251861 | -82.4018 |
| 45 | 9.251799 | -82.4018 |
| 46 | 9.209082 | -82.3702 |
| 47 | 9.205893 | -82.3613 |
| 48 | 9.20405 | -82.3586 |
| 49 | 9.204376 | -82.3495 |
| 50 | 9.148006 | -82.3188 |
| 51 | 9.144342 | -82.304 |
| 52 | 9.142509 | -82.3039 |
| 53 | 9.117063 | -82.2905 |
| 54 | 9.103291 | -82.2959 |
| 55 | 9.097376 | -82.2894 |
| 56 | 9.087325 | -82.2892 |
| 57 | 9.080062 | -82.2864 |
| 58 | 9.047563 | -82.3025 |
| 59 | 8.975792 | -82.2114 |
| 60 | 8.972712 | -82.2093 |
| 61 | 8.888629 | -82.1821 |
| 62 | 8.886136 | -82.1793 |
| 63 | 8.817734 | -82.1838 |
| 64 | 8.803599 | -82.1864 |
| 65 | 8.779725 | -82.1929 |
| 66 | 8.780231 | -82.2062 |
| 67 | 8.711039 | -82.2328 |
| 68 | 8.704428 | -82.2311 |
| 69 | 8.692706 | -82.2297 |
| 70 | 8.690358 | -82.2283 |
| 71 | 8.68742 | -82.2288 |
| 72 | 8.688723 | -82.2273 |
| 73 | 8.68526 | -82.228 |
| 74 | 8.680023 | -82.2247 |
| 75 | 8.679408 | -82.2226 |
| 76 | 8.663616 | -82.2139 |
| 77 | 8.655865 | -82.2119 |
| 78 | 8.639667 | -82.2119 |
| 79 | 8.662952 | -82.2126 |
| 80 | 8.909455 | -82.1872 |
| 81 | 8.85430 | -82.1825 |
| 82 | 9.22790 | -82.3808 |
| 83 | 9.12610 | -82.2996 |
| 84 | 9.3224 | -82.4732 |
| 85 | 8.9070 | -82.1867 |
| 86 | 9.2102 | -82.3705 |
| 87 | 9.3880 | -82.4858 |
