# Supplementary material for: Ensemble frequency ratio-maximum entropy landslide susceptibility modelling for climate‑resilient road corridors in Bocas del Toro and Chiriquí, Panama
Source: PLoS One. 2026 Nov 5;21(11):e0362084. doi: 10.1371/journal.pone.0362084 (PMC13739119; doi:10.1371/journal.pone.0362084)
Supplement: Supplementary file 2 [file pone.0362084.s002.docx]

S2 Table: Frequency ratios for the 65% and 80% training partitions

| **Factors** | **Class** | **Area (Km^2^)** | **65% Training Partition** | | | **80% Training Partition** | | |
| --- | --- | --- | --- | --- | --- | --- | --- | --- |
|  |  |  | **Landslide in class** | **% of landslide** | **FR** | **Landslide in class** | **% of landslide** | **FR** |
| Aspect | Flat | 198.22 | 0 | 0 | 0 | 2 | 2.86 | 0.18 |
|  | North | 85.75 | 9 | 15.79 | 2.27 | 10 | 14.29 | 2.05 |
|  | North East | 165.52 | 15 | 26.32 | 1.96 | 17 | 24.29 | 1.81 |
|  | East | 126.63 | 3 | 5.26 | 0.51 | 6 | 8.57 | 0.83 |
|  | South East | 126.05 | 4 | 7.02 | 0.69 | 5 | 7.14 | 0.7 |
|  | South | 123.34 | 5 | 8.77 | 0.88 | 6 | 8.57 | 0.86 |
|  | South West | 130.01 | 9 | 15.79 | 1.5 | 10 | 14.29 | 1.35 |
|  | West | 103.05 | 7 | 12.28 | 1.47 | 7 | 10 | 1.2 |
|  | North West | 119.36 | 4 | 7.02 | 0.72 | 6 | 8.57 | 0.88 |
|  | North | 54.01 | 1 | 1.75 | 0.4 | 1 | 1.43 | 0.18 |
| Elevation (m) | 5 – 129.99 | 768.69 | 34 | 59.65 | 0.96 | 41 | 58.57 | 0.94 |
|  | 129.99 – 364.34 | 277.66 | 11 | 19.30 | 0.86 | 14 | 20 | 0.89 |
|  | 364.34 -770.55 | 83.81 | 1 | 1.75 | 0.26 | 1 | 1.43 | 0.21 |
|  | 770.55 – 1,208.01 | 58.71 | 7 | 12.28 | 2.58 | 8 | 11.43 | 2.4 |
|  | 1,208.01 – 1,997 | 43.06 | 4 | 7.02 | 2.01 | 6 | 8.57 | 2.45 |
| Distance from Faultline (m) | 0 – 2,482.82 | 526.57 | 24 | 42.11 | 0.99 | 28 | 40 | 0.94 |
|  | 2,482.82 – 5,763.69 | 281.06 | 10 | 17.54 | 0.77 | 10 | 14.29 | 0.63 |
|  | 5,763.69 -9,931.29 | 233.95 | 6 | 10.53 | 0.55 | 8 | 11.43 | 0.6 |
|  | 9,931.29 -15,340.29 | 98.56 | 7 | 12.28 | 1.53 | 8 | 11.43 | 1.43 |
|  | 15,340.29 – 22,611.41 | 91.78 | 10 | 17.54 | 2.35 | 16 | 22.86 | 3.07 |
| Distance from river | 0 – 169.2 | 360.75 | 19 | 33.33 | 1.14 | 22 | 31.43 | 1.07 |
|  | 169.2 – 372.24 | 364.04 | 19 | 33.33 | 1.13 | 25 | 35.71 | 1.21 |
|  | 372.24 – 592.21 | 284.66 | 14 | 24.56 | 1.06 | 17 | 24.29 | 1.05 |
|  | 592.21 – 888.31 | 178.02 | 4 | 7.02 | 0.49 | 5 | 7.14 | 0.49 |
|  | 888.31 – 2,157.33 | 44.46 | 1 | 1.75 | 0.49 | 1 | 1.43 | 0.4 |
| Geology | Alluvium/mangrove corals/ coalcorals/conglomerates | 549.87 | 12 | 21.05 | 0.47 | 17 | 24.29 | 0.54 |
|  | Shale/sandstone | 53.65 | 1 | 1.75 | 0.4 | 1 | 1.43 | 0.33 |
|  | Andesite/basalt/ intrusives | 286.99 | 20 | 35.09 | 1.51 | 24 | 34.29 | 1.47 |
|  | Shale/limestone/clay | 0.01 | 0 | 0 | 0 | 0 | 0 | 0 |
|  | Sandstone/shale/siltstone | 66.49 | 3 | 5.26 | 0.98 | 3 | 4.29 | 0.79 |
|  | Basalt/andesite/ash | 0.07 | 0 | 0 | 0 | 0 | 0 | 0 |
|  | Interbedded andesite/ limestone/shale | 20.27 | 0 | 0 | 0 | 0 | 0 | 0 |
|  | Granodiorites/Monzonites | 7.47 | 0 | 0 | 0 | 0 | 0 | 0 |
|  | Pyroclast/conglomerates/ sandstone/shale | 247.11 | 23 | 40.35 | 2.01 | 25 | 35.71 | 1.78 |
| Geomorphon | Flat | 70.25 | 0 | 0 | 0 | 0 | 0 | 0 |
|  | Peak | 19.73 | 0 | 0 | 0 | 0 | 0 | 0 |
|  | Ridge | 103.58 | 1 | 1.75 | 0.21 | 1 | 1.43 | 0.17 |
|  | Shoulder | 14.21 | 0 | 0 | 0 | 0 | 0 | 0 |
|  | Spur | 186.47 | 13 | 22.81 | 1.51 | 15 | 21.43 | 1.42 |
|  | Slope | 458.03 | 33 | 57.89 | 1.56 | 41 | 58.57 | 1.58 |
|  | Hollow | 145.07 | 5 | 8.77 | 0.74 | 6 | 8.57 | 0.73 |
|  | Foot slope | 89.03 | 0 | 0 | 0 | 0 | 0 | 0 |
|  | Valley | 141.19 | 5 | 8.77 | 0.77 | 7 | 10 | 0.87 |
|  | Pit | 4.35 | 0 | 0 | 0 | 0 | 0 | 0 |
| LS-factor | 0 – 5.02 | 883.66 | 20 | 35.09 | 0.49 | 26 | 37.14 | 0.52 |
|  | 5.02 – 17.56 | 285.89 | 34 | 59.65 | 2.57 | 38 | 54.29 | 2.34 |
|  | 17.56 – 42.65 | 54.67 | 3 | 5.26 | 1.19 | 6 | 8.57 | 1.93 |
|  | 42.65 – 100.35 | 6.9 | 0 | 0 | 0 | 0 | 0 | 0 |
|  | 100.35 – 639.71 | 0.82 | 0 | 0 | 0 | 0 | 0 | 0 |
| NDVI | -0.99 - -0.080 | 5.88 | 0 | 0 | 0 | 0 | 0 | 0 |
|  | -0.08. – 0.43 | 18.57 | 2 | 3.51 | 2.33 | 2 | 2.86 | 1.9 |
|  | 0.43 – 0.69 | 88.89 | 13 | 22.81 | 3.16 | 15 | 21.43 | 2.97 |
|  | 0.69 – 0.82 | 295.52 | 21 | 36.84 | 1.54 | 31 | 45.71 | 1.85 |
|  | 0.82 – 0.94 | 823.07 | 21 | 36.84 | 0.55 | 22 | 31.43 | 0.47 |
| Plan Curvature | 14.35 - - 0.41 | 239.87 | 16 | 28.07 | 1.44 | 18 | 25.71 | 1.32 |
|  | -0.41 - -0.11 | 88.74 | 5 | 8.77 | 1.22 | 7 | 10 | 1.39 |
|  | -0.11 - -0.01 | 27.09 | 4 | 7.02 | 3.19 | 5 | 7.14 | 3.25 |
|  | -0.01- 0.58 | 671.17 | 16 | 28.07 | 0.52 | 21 | 30 | 0.55 |
|  | 0.58 – 11.04 | 205.05 | 16 | 28.07 | 1.69 | 19 | 27.14 | 1.63 |
| Profile curvature | -17.14 - - 0.62 | 228.85 | 5 | 8.77 | 0.47 | 7 | 10 | 0.54 |
|  | -0.62 - - 0.20 | 117.91 | 5 | 8.77 | 0.92 | 6 | 8.57 | 0.90 |
|  | - 0.20 - - 0.06 | 59.61 | 2 | 3.51 | 0.73 | 2 | 2.86 | 0.59 |
|  | -0.06 – 0.64 | 684.87 | 22 | 38.6 | 0.69 | 29 | 41.43 | 0.75 |
|  | 0.64 – 18.56 | 140.69 | 23 | 40.35 | 3.53 | 26 | 37.14 | 3.25 |
| Rainfall (extreme) | 263.85 – 317.6 | 391.23 | 17 | 29.82 | 0.94 | 17 | 24.29 | 0.76 |
|  | 317.6 – 349.49 | 440.49 | 28 | 49.12 | 1.37 | 38 | 54.29 | 1.52 |
|  | 349.49 – 384.11 | 175.82 | 5 | 8.77 | 0.61 | 7 | 10 | 0.7 |
|  | 384.11 – 422.38 | 161.31 | 4 | 7.02 | 0.54 | 4 | 5.71 | 0.44 |
|  | 422.38 – 496.17 | 63.08 | 3 | 5.26 | 1.03 | 4 | 5.71 | 1.12 |
| Soil depth (cm) | 0 – 2,426.18 | 570.17 | 40 | 70.18 | 1.52 | 51 | 72.86 | 1.57 |
|  | 2,426.18 – 4,270.07 | 447.16 | 15 | 26.32 | 0.72 | 17 | 24.29 | 0.67 |
|  | 4,270.07 – 9,607.66 | 101.39 | 2 | 3.51 | 0.43 | 2 | 2.86 | 0.35 |
|  | 9,607.66 – 15,624.58 | 31.13 | 0 | 0 | 0 | 0 | 0 | 0 |
|  | 15,624.58 – 24,747.0 | 82.08 | 0 | 0 | 0 | 0 | 0 | 0 |
| SPI | -4.26 - -0.98 | 240.64 | 2 | 3.51 | 0.18 | 2 | 2.86 | 0.15 |
|  | -0.98 - -0.09 | 148.53 | 8 | 14.04 | 1.16 | 10 | 14.29 | 1.18 |
|  | -0.09 – - 0.03 | 11.33 | 1 | 1.75 | 1.91 | 1 | 1.42 | 1.55 |
|  | - 0.03 – 1.11 | 641.53 | 32 | 56.14 | 1.08 | 40 | 57.14 | 1.1 |
|  | 1.11 – 11.84 | 189.9 | 14 | 24.56 | 1.59 | 17 | 24.29 | 1.58 |
| Slope | 0 – 5.09 | 532.54 | 2 | 3.51 | 0.08 | 4 | 5.71 | 0.13 |
|  | 5.09 – 12.16 | 271.72 | 8 | 14.04 | 0.64 | 11 | 15.71 | 0.71 |
|  | 12.16 – 19.80 | 219.49 | 19 | 33.33 | 1.87 | 20 | 28.57 | 1.6 |
|  | 19.80 – 29.13 | 151.21 | 23 | 40.35 | 3.29 | 26 | 37.14 | 3.03 |
|  | 29.13 – 72.13 | 56.97 | 5 | 8.77 | 1.9 | 9 | 12.86 | 2.78 |
| TWI | 1.56 – 5.43 | 452.59 | 34 | 59.65 | 1.62 | 42 | 60 | 1.63 |
|  | 5.43 – 7.37 | 442.47 | 20 | 35.09 | 0.98 | 22 | 31.43 | 0.88 |
|  | 7.37 – 9.53 | 178.72 | 3 | 5.26 | 0.36 | 4 | 5.71 | 0.39 |
|  | 9.53 – 12.58 | 129.93 | 0 | 0 | 0 | 1 | 1.42 | 0.14 |
|  | 12.58 – 20.55 | 28.22 | 0 | 0 | 0 | 1 | 1.42 | 0.62 |
