# Supplementary material for: Ensemble frequency ratio-maximum entropy landslide susceptibility modelling for climate‑resilient road corridors in Bocas del Toro and Chiriquí, Panama
Source: PLoS One. 2026 Nov 5;21(11):e0362084. doi: 10.1371/journal.pone.0362084 (PMC13739119; doi:10.1371/journal.pone.0362084)
Supplement: Supplementary file 3 [file pone.0362084.s003.docx]

S3 Table: AUCs Standard Errors and Confident Intervals for the 65% and 80% sensitivity scenarios

| **Scenario** | **Model** | **Success Rate** | | **Prediction Rate** | |
| --- | --- | --- | --- | --- | --- |
|  |  | **Std. Error** | **95% Confident Interval  (Lower–Upper)** | **Std. Error** | **95% Confident Interval  (Lower–Upper)** |
| 65:35% Partition | FR | 0.014 | 0.886-0.939 | 0.031 | 0.775-0.896 |
|  | MaxEnt | 0.015 | 0.893-0.952 | 0.031 | 0.813-0.936 |
|  | Ensemble | 0.014 | 0.905-0.959 | 0.029 | 0.824-0.937 |
| 80:20% Partition | FR | 0.015 | 0.870-0.927 | 0.034 | 0.785-0.920 |
|  | MaxEnt | 0.015 | 0.885-0.945 | 0.028 | 0.854-0.964 |
|  | Ensemble | 0.013 | 0.899-0.950 | 0.029 | 0.845-0.959 |
