# Supplementary material for: Ensemble frequency ratio-maximum entropy landslide susceptibility modelling for climate‑resilient road corridors in Bocas del Toro and Chiriquí, Panama
Source: PLoS One. 2026 Nov 5;21(11):e0362084. doi: 10.1371/journal.pone.0362084 (PMC13739119; doi:10.1371/journal.pone.0362084)
Supplement: Supplementary file 4 [file pone.0362084.s004.docx]

Table S4: Relative proportions of observed landslides within each model’s susceptibility class for the sensitivity scenarios

| **Training Scenario** | **Susceptibility** | **FR Model** | | | **MaxEnt Model** | | | **AUC Ensemble Model** | | |
| --- | --- | --- | --- | --- | --- | --- | --- | --- | --- | --- |
|  |  | **Area (km^2^)** | **F** | **%** | **Area (km^2^)** | **F** | **%** | **Area (km^2^)** | **F** | **%** |
| 65:35%  Partition | Very low | 214.54 | 0 | 0 | 722.29 | 6 | 6.9 | 431.26 | 0 | 0 |
|  | Low | 296.59 | 0 | 0 | 244.81 | 6 | 6.9 | 378.79 | 5 | 5.75 |
|  | Moderate | 300.99 | 7 | 8.05 | 142.55 | 16 | 18.39 | 235.21 | 10 | 11.49 |
|  | High | 271.94 | 21 | 24.14 | 79.13 | 18 | 20.69 | 127.64 | 20 | 22.99 |
|  | Very high | 147.87 | 59 | 67.81 | 43.15 | 41 | 47.13 | 59.03 | 52 | 59.77 |
|  | **TOTAL** | **1231.93** | **87** | **100** | **1231.93** | **87** | **100** | **1231.93** | **87** | **100** |
| 80:20%  Partition | Very low | 226.84 | 0 | 0 | 696.06 | 3 | 3.45 | 420.51 | 0 | 0 |
|  | Low | 300.11 | 1 | 1.15 | 272.73 | 8 | 9.2 | 381.61 | 3 | 3.45 |
|  | Moderate | 303.52 | 7 | 8.05 | 150.23 | 17 | 19.54 | 250.83 | 13 | 14.94 |
|  | High | 264.98 | 23 | 26.44 | 73.22 | 15 | 17.24 | 126.65 | 23 | 26.44 |
|  | Very high | 136.48 | 56 | 64.37 | 39.69 | 44 | 50.57 | 52.33 | 48 | 55.17 |
|  | **TOTAL** | **1231.93** | **87** | **100** | **1231.93** | **87** | **100** | **1231.93** | **87** | **100** |
