# Supplementary material for: Ensemble frequency ratio-maximum entropy landslide susceptibility modelling for climate‑resilient road corridors in Bocas del Toro and Chiriquí, Panama
Source: PLoS One. 2026 Nov 5;21(11):e0362084. doi: 10.1371/journal.pone.0362084 (PMC13739119; doi:10.1371/journal.pone.0362084)
Supplement: Supplementary file 5 [file pone.0362084.s005.docx]

**b**

**a**

**d**

**c**

S1 Fig: ROC-AUCs success and prediction rates for the 65:35% and 80:20% inventory allocation scenarios
