# Supplementary material for: Ensemble frequency ratio-maximum entropy landslide susceptibility modelling for climate‑resilient road corridors in Bocas del Toro and Chiriquí, Panama
Source: PLoS One. 2026 Nov 5;21(11):e0362084. doi: 10.1371/journal.pone.0362084 (PMC13739119; doi:10.1371/journal.pone.0362084)
Supplement: Supplementary file 7 [file pone.0362084.s007.docx]

S3 Fig: Frequency Ratio, Maximum Entropy and Ensemble landslide susceptibility patterns for the 80% training partition scenario
